# Dendritic Cell-Based Immunotherapy in Lung Cancer

**DOI:** 10.3389/fimmu.2020.620374

**Published:** 2021-02-12

**Authors:** Dieter Stevens, Joline Ingels, Sandra Van Lint, Bart Vandekerckhove, Karim Vermaelen

**Affiliations:** ^1^ Respiratory Medicine - Thoracic Oncology Cluster, Ghent University Hospital, Ghent, Belgium; ^2^ Respiratory Medicine - Tumor Immunology Laboratory, Ghent University, Ghent, Belgium; ^3^ Department of Diagnostic Sciences, Ghent University Hospital, Ghent, Belgium; ^4^ GMP Cell Therapy Unit, Department of Regenerative Medicine, Ghent University Hospital, Ghent, Belgium; ^5^ Cancer Research Institute Ghent, Ghent University, Ghent, Belgium

**Keywords:** dendritic cell, cancer vaccine, lung cancer, immunotherapy, tumor antigen, immune checkpoint blockade

## Abstract

Lung cancer remains the leading cause of cancer-related death worldwide. The advent of immune checkpoint inhibitors has led to a paradigm shift in the treatment of metastatic non-small cell and small cell lung cancer. However, despite prolonged overall survival, only a minority of the patients derive clinical benefit from these treatments suggesting that the full anti-tumoral potential of the immune system is not being harnessed yet. One way to overcome this problem is to combine immune checkpoint blockade with different strategies aimed at inducing or restoring cellular immunity in a tumor-specific, robust, and durable way. Owing to their unique capacity to initiate and regulate T cell responses, dendritic cells have been extensively explored as tools for immunotherapy in many tumors, including lung cancer. In this review, we provide an update on the nearly twenty years of experience with dendritic cell-based immunotherapy in lung cancer. We summarize the main results from the early phase trials and give an overview of the future perspectives within this field.

## Introduction

Lung cancer is the leading cause of cancer-related mortality worldwide, with 1.8 million deaths estimated in 2018 (1). Non-small cell lung cancer (NSCLC) represents 85% of all cases, while small cell lung cancer (SCLC) accounts for approximately 15% of all lung cancers. Treatment depends on tumor stage at diagnosis and comprises surgery, radiotherapy and chemotherapy in early stages, and palliative treatments in metastatic disease. Since almost three quarter of the patients are diagnosed with stage III or IV disease and a significant number of patients relapse systemically after a curative treatment, prognosis remains poor with an estimated 5-year overall survival (OS) of only 18% (2).

Immune checkpoint blockade with drugs that target the programmed cell death protein pathway (PD-1/PD-L1) has changed the therapeutic landscape of locally advanced and metastatic lung cancer. Several randomized controlled trials have shown promising results with checkpoint inhibitors alone (3), or in combination with chemotherapy (4–7). PD-1/PD-L1 inhibitors such as nivolumab, pembrolizumab and atezolizumab are now approved for the first and second line treatment of metastatic NSCLC (without actionable driver mutations) and SCLC, and as a maintenance treatment after chemoradiotherapy in inoperable stage III NSCLC (durvalumab). In addition, trials with checkpoint inhibitors as adjuvant or neo-adjuvant therapy in resectable lung cancer are now underway and the results are eagerly awaited.

Despite better outcomes in terms of OS, only a minority of the patients derive clinical benefit from these treatments. In metastatic NSCLC, more than 40% to 50% of the subjects do not respond to immune checkpoint blockade when given in the first line (3, 4, 6). In the second or higher line setting, the objective response rate (ORR) is even lower (< 20%) (8–11). These results suggest that the full anti-tumoral potential of the immune system is not being harnessed yet, possibly explained by immune evasion mechanisms developed by the tumor to escape from immune destruction (12, 13). One way to overcome this problem is to combine immune checkpoint inhibition with other strategies aimed at inducing or restoring cellular immunity such as cancer vaccination (14, 15).

The goal of therapeutic cancer vaccines is to instruct the patient’s own immune system to kill cancer cells and to induce immunological memory against later disease relapse (16–18). In contrast to immune checkpoint blockade, which impacts the full T cell repertoire including self-reactive lymphocytes which translates into substantial toxicity, cancer vaccines expose the patient’s immune system to a unique selection of relevant antigenic targets resulting in a highly tumor-focused immune response (17, 18). A limitation of this strategy is that the ability of such vaccines to activate patient’s T cells depends on the characteristics and level of activation of local dendritic cells (DCs), which are frequently dysfunctional in patients with advanced cancer (17). Hence, vaccines by themselves have failed to show any clinical benefit in NSCLC so far (19–23).

Cell-based approaches that involve patient’s *ex vivo*-generated antigen presenting cells (APCs) such as DC-based vaccines avoid the reliance on endogenous APCs and are nowadays one of the most advanced forms of cancer immunotherapy (17). DCs, first identified by Ralph Steinman in 1973 (24), are recognized as the most potent APCs and play a pivotal role in the initiation, programming, and regulation of tumor-specific immune responses (25, 26). They are seeded in all tissues and continuously sample their environment for danger signals and antigens such as those derived from evolving cancer cells. DCs are unique in initiating *de novo* immune responses by processing the captured antigen to peptides and presenting these peptides to naive T cells in lymphoid tissues on major histocompatibility complex (MHC) molecules (26, 27).

**Figure 1:**
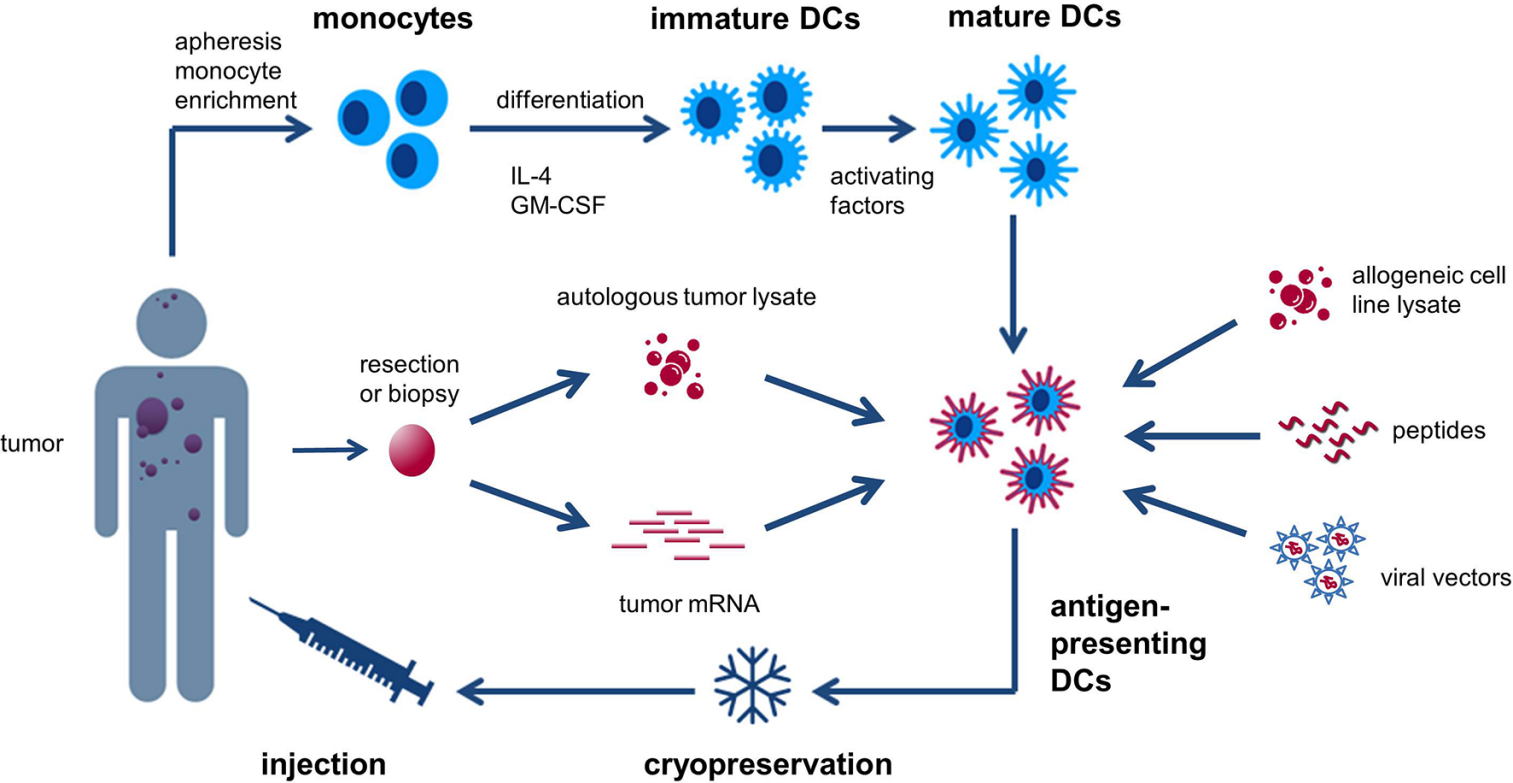
Generic recipe of classical monocyte-derived dendritic cells (DCs). Monocytes are obtained from the patient’s peripheral blood and cultured with IL-4 and GM-CSF to generate immature DCs. These cells are subsequently exposed to activating factors for maturation and loaded with tumor-associated antigens (TAAs). The antigen-loaded DCs are then cryopreserved and injected back into the patient. Different sources of TAAs can be used and include cancer cell line lysate, whole tumor lysate, tumor-derived peptides, (synthetic) protein antigen(s), mRNA(s) encoding selected tumor antigen(s), autologous whole-tumor-derived mRNA, or antigens packaged within viral vectors.

Classical DC-based “vaccines” consist of DCs derived *in vitro* from autologous peripheral blood monocytes (PBMCs), exposed to activating factors for maturation and subsequently loaded with tumor-associated antigens (TAAs) (**Figure 1**) (28). These cells are then injected into the patient, a process that has been repeatedly shown to be safe and feasible (27, 29). Alternatively, naturally circulating DCs can be isolated and activated thereby avoiding lengthy *ex vivo* culture periods (30). The selection of tumor antigens for loading onto DCs is crucial to maximize the likelihood of eliciting a strong and tumor-directed immune response. Different sources of TAAs can be used and include cancer cell line lysate, whole tumor lysate, tumor-derived peptides, (synthetic) protein antigen(s), mRNA(s) encoding selected tumor antigen(s), autologous whole-tumor-derived mRNA or antigens packaged within viral vectors (18, 29, 31).

## Clinical Use of DCs in Oncology: Track Record and Critical Factors

In the field of cancer medicine, DC vaccination has been extensively studied in melanoma patients, as well as in patients with prostate cancer, glioma and renal cell carcinoma, with a favorable safety profile (i.e., no grade 3 or 4 toxicities), but with an ORR that seldom exceeds 15% (32–34). Paradoxically, findings from early-phase trials indicate that DC vaccination might improve survival, advocating implementation of alternative surrogate endpoints to assess the therapeutic effectiveness of DC-based immunotherapy (32).

Still, a major gap exists between the large amount of preclinical data on the exceptional immunogenic power of DCs, and the modest clinical effects in treated cancer patients. The evolving insights into the complex biology of the DC system confront us with a staggering list of parameters that should be adjusted in order to achieve optimal clinical usability. These parameters not only relate to “tweakable” biological properties of the cells, but also to more down-to-earth aspects such as route of administration, dose and frequency of administration, integration into a combinatorial approach, manufacturing, distribution logistics, and costs.

Perhaps one of the most critical factors in DC therapy, yet least systematically investigated is the choice of antigenic targets. This component varies considerably between clinical studies within the same cancer indication, with antigen selections largely made empirically in absence of any solid underlying rationale. Cancer antigens fall into the following different classes: 1) mutated antigens or neo-antigens originating from genomic alterations in cancer cells (single-nucleotide variations and indels), 2) cancer-germline (formerly cancer-testis) antigens whose expression is epigenetically suppressed in normal tissues except for gonadal cells, placenta and many cancers, 3) “differentiation” antigens, which are self-proteins shared between the cancer and the normal tissue from where it originated (e.g., CD20, Melan-A, PSA, CEA), 4) overexpressed shared antigens, which are present in normal tissues and aberrantly overexpressed in tumor cells (e.g., HER2, survivin, WT1), and finally viral oncoproteins, which are expressed in certain virus-induced cancers (e.g., HPV-E6/E7, EBV LMP-1). In addition, some tumor antigens derive their immunogenicity by means of aberrant post-translational modifications, as is the case for the MUC-1 glycoprotein where the tumor-restricted form is strongly hypo-glycosylated. Of all these categories, neo-antigens, cancer-germline antigens and viral oncoproteins are the most attractive targets for DC-based immunotherapy given the highest cancer-restricted expression, and the fact that the natural T cell repertoire has not been tolerized against them. Regardless of the type of antigen used, a major caveat is that studies or databases documenting mRNA expression in a given tumor often do not provide information on protein levels. Moreover, protein expression does not guarantee adequate presentation of antigen-derived peptides on MHC molecules, and if presented, whether these peptides will find a corresponding T cell repertoire with sufficient affinity.

Lung cancer (both NSCLC and SCLC), being a textbook example of a carcinogen-induced tumor, frequently features a high tumor mutational burden, offering opportunities for neoantigen-targeted vaccination approaches. Lung cancers are also rich in cancer-germline antigens [e.g., MAGE-A3 (22)], a number of differentiation antigens (e.g., CEA), and overexpressed shared antigens (e.g., survivin, WT1, MUC1), all being present in variable amounts across different patients. Viral oncoproteins are typically absent in human lung cancers. As we will discuss in the next section, the large majority of completed DC therapy trials in lung cancer made use of shared or tumor-associated antigens (TAAs), a few of the studies also incorporated cancer-germline antigens, and none of the published reports have described a patient-individualized neoantigen approach to date.

## DC-Based Immunotherapies in Lung Cancer

In lung cancer, the role of DC-based immunotherapy has yet to be defined. Since the early 2000s, several, mostly non- randomized clinical trials (RCTs) with DC immunotherapy have been conducted, each typically involving a small number of patients and very heterogeneous designs. Over the same timeframe, the lung cancer therapeutic landscape has experienced dramatic changes, with the emergence of oncogene-targeted small molecules, and later immune checkpoint inhibitors. In this review we aim to give an overview of these DC therapy trials which we categorized into four parts: DC therapy in NSCLC, DC/CIK cell therapy in NSCLC, AKT-DC therapy in NSCLC, and DC therapy in SCLC (**Tables 1** and **2**). We will examine the clinical and immunological outcome as well as safety of DC-based immunotherapy in lung cancer, while also discussing the potential challenges of the different vaccine approaches such as the choice of antigens and DC subset, the use of adjuvants and the route, dose and frequency of administration. Next, we will give some future perspectives in how DCs might be used in clinical practice.

**Table 1:** Overview of trials with dendritic cell (DC)-based immunotherapy in lung cancer.

Reference	Study population	N	Trial phase	DC subset	Maturation factors	Antigen selection and formulation	Vaccination protocol
***DC therapy in NSCLC***							
Fong et al. (35)	Metastatic CRC or NSCLC expressing CEA	12 (2 with NSCLC)	Phase 1	Flt3-mobilized circulating DCs Mature	N/A	CEA peptide	a) 2 vaccinations b) 1-month interval c) i.v. injection d) Maximum dose of 10^9^ cells per vaccination
Itoh et al. (36)	Metastatic digestive tract or lung cancer expressing CEA	10 (2 with lung cancer)	Phase 1	moDCs Immature	N/A	CEA peptide	a) 10 vaccinations b) 2-week interval c) i.d. and s.c. injection at the same site in the inguinal region d) Total dose of 2.7 × 10^7^ cells to 1.6 × 10^8^ cells e) IFN-α and TNF-α
Nair et al. (37)	Metastatic cancer	3 (1 with NSCLC)	Phase 1	moDCs Immature	N/A	CEA RNA or autologous tumor RNA	a) 4 vaccinations b) 4-week interval c) i.v. and i.d. d) 3 × 10^6^ cells (i.v.) and 1 × 10^6^ cells (i.d.) per vaccination
Kontani et al. (38)	Advanced or metastatic breast or lung cancer	14 (8 with lung cancer)	Phase 1	moDCs Mature	N/A	MUC1 antigen or tumor lysate	a) 3 to 12 vaccinations b) 2-week interval c) s.c. or intrapleural d) 4–10 × 10^6^ cells per vaccination
Hirschowitz et al. (39, 40)	Stage I-IIIB NSCLC	16	Phase 1	moDCs Mature	DCTCMF IFN-γ	Apoptotic bodies of an allogeneic NSCLC cell line that overexpressed HER2/neu, CEA, WT1, MAGE-2, and survivin	a) 2 vaccinations b) 1-month interval c) i.d. injection in the thigh d) Average dose of 9.1 × 10^7^ and 8.2 × 10^7^ cells per vaccination respectively
Ueda et al. (41)	Metastatic gastrointestinal or lung adenocarcinoma expressing CEA	18 (5 with lung cancer)	Phase 1	moDCs Immature	N/A	CEA peptide	a) Median number of 9 vaccinations (range, 5–39) b) 2-week interval c) i.d. and s.c. injection at the same site in the inguinal region d) 0.5–5 × 10^7^ cells per vaccination
Chang et al. (42)	Stage IV NSCLC with malignant pleural effusion	8	Phase 1	moDCs Mature	TNF-α	Tumor cell lysate derived from malignant pleural effusion specimens	a) 6 vaccinations b) 1-week interval for the first 4 vaccinations, then twice biweekly c) i.n. injection under sonographic guidance d) Dose not mentioned
Morse et al. (43)	Metastatic cancer expressing CEA	14 (3 with NSCLC)	Phase 1	moDCs Immature	N/A	Fowlpox virus encoding CEA and a triad of costimulatory molecules (rF-CEA(6D)-TRICOM)	a) 4 (first cohort) or 8 vaccinations (second cohort) b) triweekly c) s.c. and i.d. injection in the same limb d) Dose not mentioned
Hirschowitz et al. (44)	Stage I-IIIB NSCLC	14	Phase 1	moDCs Immature	N/A	Apoptotic bodies of an allogeneic NSCLC cell line that overexpressed HER2/neu, CEA, WT1, MAGE-2, and survivin	a) 2 vaccinations b) 1-month interval c) i.d. injection in the thigh d) Average dose of 8.2 × 10^7^ and 7.9 × 10^7^ cells per vaccination respectively
Mayordomo J et al. (45)	Metastatic cancer	15 (2 with NSCLC)	Phase 1	moDCs Immature	N/A	Autologous tumor lysate	a) 3 vaccinations b) 3-week interval c) i.v. injection d) Median dose of 6.2 × 10^7^ cells per vaccination e) IL-2, INF-α and GM-CSF
Um et al. (46)	Stage IIIB-IV NSCLC	15	Phase 1	moDCs Mature	TNF-α IL-1 IL-6 PGE2	Autologous tumor lysate	a) 3 vaccinations and after verification of tolerability 2 subsequent vaccinations b) 2-week interval for the first 3 vaccinations and 1-month interval for the subsequent 2 vaccinations c) i.d. injection d) Maximum dose of 12 × 10^6^ cells per vaccination
Perroud et al. (47)	Stage IIIB-IV NSCLC	5	Phase 1	moDCs Mature	IFN-γ	WT1 peptide CEA peptide MAGE-1 peptide HER-2 peptide	a) 2 vaccinations b) 2-week interval c) s.c. and i.v. injection in separate arms d) 5 × 10^7^ cells per vaccination
Takahashi et al. (48)	Locally advanced or metastatic NSCLC	62	Retrospective analysis	moDCs Mature	OK-432 PGE2	Autologous tumor lysate or peptide antigens (WT1, MUC1, CEA) according to the HLA-A pattern.	a) Median number of 10 vaccinations (range, 4–31) b) Biweekly c) i.d. injection near the axillar and/or inguinal lymph nodes d) 1 × 10^7^ cells per vaccination
Hu et al. (49)	Stage IIIB-IV NSCLC	27	Phase 1	moDCs Immature	N/A	Autologous tumor lysate	a) Number of vaccinations not mentioned b) 3-week interval c) i.d. injection d) Average dose of 1 × 10^7^ cells per vaccination
Takahashi et al. (50)	Locally advanced or metastatic NSCLC	260	Retrospective analysis	moDCs Mature	OK-432 PGE2	WT1 peptide MUC1 peptide	a) Median number of 7 vaccinations (range, 5–34) b) Biweekly c) i.d. injection near the axillar and/or inguinal lymph nodes d) 1 × 10^7^ cells per vaccination e) OK-432
Li et al. (51)	Stage I-IIIB NSCLC	16	Phase 1	moDCs Mature	IL-1β IL-6 TNF-α IFN-γ PGE2 Poly I:C	rMAGE-3 peptide rSurvivin peptide	a) 2 vaccinations b) 1-month interval c) i.d. d) 9.1 × 10^7^ cells and 8.2 × 10^8^ cells per vaccination respectively
Lee et al. (52)	Stage III-IV NSCLC	16	Phase 1	moDCs Immature	N/A	Viral vector (Ad.CCL21-DC)	a) 2 vaccinations b) 1-week interval c) CT- or bronchoscopy guided i.t. injection d) Maximum dose of 3 × 10^7^ cells per vaccination
Teramoto et al. (53)	Stage IIIB-IV NSCLC	40	Retrospective analysis	moDCs Mature	OK-432	MUC1 peptide	a) range, 1–42 vaccinations b) 2-week interval c) s.c. injection d) 1 × 10^7^ cells per vaccination
Ge et al. (54)	Resected stage I-IIIA NSCLC	15	Phase 1	moDCs Mature	Flagellin SOSC1-specific small interfering RNA	MUC1 peptide Survivin peptide	a) 3 vaccinations b) 1-week interval c) i.v. injection d) 1 × 10^6^,1 × 10^7^ or 8 × 10^7^ cells per vaccination
***DC/CIK therapy in NSCLC***							
Li et al. (55)	Stage I-IIIA NSCLC	84 (42 received DC/CIK)	Phase 1/2	moDCs Mature	N/A	Autologous tumor lysate	a) 4 vaccinations b) 1-month interval c) i.v. injection d) Average dose of 13 × 10^9^ cells per vaccination
Zhong et al. (56)	Stage IIIB-IV NSCLC expressing CEA	28 (14 patients received DC/CIK)	Phase 1/2	moDCs Immature	N/A	CEA peptide	a) 4 vaccinations b) 1-month interval c) i.v. injection d) Average dose of 8.1 × 10^6^ cells per vaccination
Shi et al. (57)	Stage IIIB-IV NSCLC	60 (30 patients received DC/CIK)	RCT	moDCs Mature	GM-CSF TNF IL-6	N/A	a) 4-vaccinations b) 1-week interval c) s.c. injection d) Dose not mentioned
Yang et al. (58)	Stage IIIB-IV NSCLC	102 (61 patients received DC/CIK)	Paired cohort study	moDCs Immature	N/A	Autologous tumor lysate	a) 4 vaccinations b) 1-month interval c) i.v. injection d) Average dose of 12.5 × 10^9^ cells per vaccination
Shi et al. (59)	Stage IIIB-IV NSCLC with EGFR exon 19 and/or 21 mutation	54 (26 patients received DC/CIK)	RCT	moDCs Immature	N/A	Autologous tumor lysate	a) 8 vaccinations b) 1-week interval c) s.c. injection d) Dose not mentioned
Zhao et al. (60)	Resected stage I-III NSCLC (arm 1) or metastatic NSCLC (arm 2)	50	Phase 1	moDCs Mature	TNF-α	Human A549 or SK-MES-1 lung cancer cell lysate	a) 4 vaccinations b) 1-week interval c) s.c. (DC) and i.v. injection (DC/CIK) d) 1.5 × 10^7^ cells per vaccination
Zhu et al. (61)	Stage IIIB NSCLC	65 (30 received DC/CIK)	RCT	moDCs Mature	TNF-α	None	a) 4 vaccinations b) 3-week interval c) i.v. injection d) Dose not mentioned
Zhang et al. (62)	Stage IIIB-IV NSCLC	99	Retrospective analysis	moDCs Immature	N/A	Human SK-MES-1 and human SPC-A-1 lung cancer cell lysate	a) 6 vaccinations b) 1-week interval c) i.v. injection (first 3 doses) and i.d. injection (last 3 doses) d) 1 × 10^7^ cells per vaccination
Zhang et al. (63)	Stage III-IV NSCLC	82 (21 received DC/CIK)	Phase 2	moDCs Mature	TNF-α	MUC1 peptide	a) 4 vaccinations b) 1-week interval c) s.c. injection d) 1 × 10^7^ cells per vaccination
Song et al. (64)	Resected stage IIB-IIIA NSCLC	169	Phase 2	moDCs Mature	N/A	Human A549 or SK-MES-1 lung cancer cell lysate	a) 5 vaccinations within 2 weeks per cycle b) 12-24 week-interval (1–2 cycles) or 4–12 week interval (3–5 cycles) c) i.v. (DC/CIK) and s.c. injection (DC) d) 20 × 10^6^ cells per vaccination
Chen et al. (65)	Advanced solid tumors	37 (5 with NSCLC)	Phase 1	moDCs Mature	TNF-α	N/A	a) Median number of 12 vaccinations (range, 3–26) b) 1-week interval for the first 4 doses, then 2-week interval thereafter c) i.v. injection d) Average dose of 8.8 × 10^9^ cells per vaccination
***AKT-DC therapy in NSCLC***							
Kimura et al. (66)	Resected stage III-IV NSCLC with N2 disease	31	Phase 2	DCs obtained from tissue cultures of TDLNs Mature	N/A	N/A	a) Median number of 11 courses (range, 2–18) b) 2-month interval c) i.v. injection d) Mean dose of 7.07 × 10^9^ cells per course e) IL-2
Kimura et al. (67, 68)	Resected stage IB-IV NSCLC	103 (50 received AKT-DC)	Phase 3 RCT	DCs obtained from tissue cultures of TDLNs Mature	N/A	N/A	a) Median number of 15 courses b) 1-month interval for the first 6 months, and 2-month interval thereafter c) i.v. injection d) Mean dose of 10, 2 × 10^9^ cells per course
***DC therapy in SCLC***							
Chiappori et al. (69)	Extensive disease SCLC	54	Phase 1/2	moDCs Mature	N/A	p53 viral vector (Ad.p53)	a) 3 vaccinations and if no PD after reassessment 3 subsequent vaccinations b) 2-week interval for the first 3 vaccinations and 4-week interval for the subsequent 3 vaccinations c) i.d. injection d) Maximum dose of 5 × 10^6^ cells per vaccination
Chiappori et al. (70, 71)	Extensive disease SCLC	69 (51 received DC vaccine)	Phase 2 RCT	moDCs Mature	N/A	p53 viral vector (Ad.p53)	a) 3 vaccinations and if no PD after reassessment 3 subsequent vaccinations b) 2-week interval for the first 3 vaccinations and 4-week interval for the subsequent 3 vaccinations c) i.d. injection d) Average dose of 2–5 × 10^6^ cells per vaccination.

a) number of vaccinations.

b) dose interval.

c) route of administration.

d) number of DCs injected.

e) adjuvants used.

Ad-CCL21-DC, dendritic cells transduced with an adenoviral vector expressing the CCL21 gene; Ad.p53, dendritic cells transduced with an adenoviral vector expressing p53; anti-PD-1, anti-programmed death-1; CCL21, chemokine ligand 21; CEA, carcinoembryonic antigen; CIK cell, cytokine induced killer cell; CRC, colorectal cancer; DCTCMF, dendritic cell/T cell-derived maturation factor; GM-CSF, granulocyte-macrophage colony-stimulating factor; HER-2, human epidermal growth factor receptor 2; HLA, human leucocyte antigen; i.d., intradermal; IL, interleukin; i.n., intranodal; IFN-α, interferon-alpha; IFN-γ, interferon-gamma; i.t., intratumoral; i.v., intravenous; MAGE-1, melanoma-associated antigen-1; MAGE-2, melanoma-associated antigen-2; moDCs, monocyte derived dendritic cells; MUC1, mucin 1; N, number of patients; N/A, not applicable; NSCLC, non-small cell lung cancer; PGE2, prostaglandin E2; RCT, randomized controlled trial; rMAGE-3, recombinant melanoma-associated antigen-3; rSurvvin, recombinant Survivin; s.c., subcutaneous; TDLN, tumor-draining lymph node; TNF-α, tumor necrosis factor-alpha; WT1, wilms tumor protein l.

**Table 2:** Clinical and immunological outcomes of dendritic cell (DC)-based immunotherapy in lung cancer.

Reference	Safety	ORR*	Survival	Immune response	Comments
***DC therapy in NSCLC***					
Fong et al. (35)	Grade 1 or 2 transfusion reaction in 7 patients Grade 1 or 2 diarrhea in 5 patients	0/2 (0%)	N/A	CEA-specific CD8+ T cell immunity was seen in 7/12 patients (58.3%).	Objective responses were observed in 2 patients, both with CRC.
Itoh et al. (36)	Grade 1 local reaction at the injection site in 2 patients	0/2 (0%)	N/A	A positive response to DTH skin test was seen in 2/10 patients (20%) of which 1 patient with lung cancer. A CEA-specific CTL response was seen in 1/10 patients (10%).	A continuous decrease of serum CEA was observed in 1 patient with lung cancer. Two patients had SD (1 patient with lung cancer). Clinical and immunological responses were only observed in patients treated with adjuvant use of INF-α and TNF-α.
Nair et al. (37)	No toxicities	0/1 (0%)	N/A	A CEA specific and tumor antigen-specific CTL response was seen in the patient with NSCLC. The tumor specific immune response was greater than the CEA specific immune response.	
Kontani et al. (38)	Fever in 7 patients	2/8 (25%)	The MST was significantly longer in MUC positive patients versus MUC negative patients (16.8 vs. 3.8 months; p = 0.0101)	A positive response to DTH skin test was seen in 5/9 patients (55.6%). A MUC1-specific CTL response was seen in 3/7 patients (42.9%).	Two patients with lung cancer had a PR. The patients who acquired tumor-specific immunity responded to treatment, i.e., reduction in tumor marker levels and disappearance of malignant pleural effusions.
Hirschowitz et al. (39, 40)	No serious AEs Grade 1 local reaction at the injection site in 10 patients Grade 1 fatigue in 3 patients	N/A	N/A	An antigen-specific immune response was seen in 6/16 patients (37.5%).	Favorable and unfavorable clinical outcomes were independent of measured immunologic responses. Five patients had disease recurrence or progression of which 3 patients died of PD.
Ueda et al. (41)	No toxicities	0/5 (0%)	Survival time ranged from 3 to 46+ months with a median of 8 months.	A positive response to DTH skin test was seen in 5/11 patients (45.5%). A CEA-specific CTL response was seen in 6/11 patients (54.5%).	Three of the 5 patients with lung cancer had prolonged and/or marked decreases in serum CEA levels after therapy.
Chang et al. (42)	No grade 2 to 4 AEs	1/8 (12.5%)	N/A	Minor to moderate increases in T cell responses against tumor antigens were observed in 6/8 patients (75%).	Two patients had SD. The 2 patients who had longer disease control also had better T cell responses.
Morse et al. (43)	No grade 3 or 4 AEs	0/3 (0%)	N/A	A CEA-specific immune response among both CD4+ and CD8+ T cells was seen in all evaluable patients (100%). There was a trend for a greater peak frequency of CEA-specific T cells among those with either a minor response or SD.	One patient had a significant decrease in the CEA level and a minor regression in a retroperitoneal and supraclavicular adenopathy. Five other patients were stable trough at least one cycle of immunization.
Hirschowitz et al. (44)	Local reactions at the injection site in most subjects (not specified)	N/A	N/A	A clear immune response was seen in 9/14 patients (64.2%).	Five patients had disease recurrence or progression of which 3 patients died of progressive disease. Three of 5 patients with PD showed no immunological response.
Mayordomo et al. (45)	Fever in 6 patients Asthenia in 11 patients	0/2 (0%)	Survival time ranged from 0 to 29+ months with a median of 7 months.	A positive response to DTH skin test was seen in 9/15 patients (60%)	Seven patients, of which 1 with NSCLC, had SD for > 3 months, and 7 other patients had PD. Time to progression ranged from 0 to 10 months with a median of 3 months.
Um et al. (46)	No grade 3 or 4 AEs Grade 1 fever in 1 patient	0/8 (0%)	N/A	An increase in the percentage of CD8+ cells expressing INF-γ was seen in 5/9 patients (55.6%).	There were mixed responses that fulfill PD definition but demonstrated some clinical benefit in 2 patients.
Perroud et al. (47)	Grade 2 fatigue and chills in 1 patient	N/A	Survival time ranged from 82 to 277 days from the last dose of the vaccine with a median of 112 days.	An improvement in the specific immune response after immunization was seen in all patients (100%) but was short lasting.	From the last dose of the vaccine, the time to disease progression ranged between 1 and 82 days.
Takahashi et al. (48)	No serious AEs Grade 1 fever in 13 patients Grade 2 fever in 2 patients Grade 1 local reaction at the injection site in 26 patients	5/62 (8.1%)	MST = 12 months	N/A	Standard chemotherapy regimens were continued in 36 patients during DC vaccination. DCR was seen in 50% of the patients. Better survival was found in patients with ECOG-PS 0 or 1 and in patients who received > 5 vaccinations.
Hu et al. (49)	Grade 1 fever in 5 patients	3/27 (11.1%)	Median OS = 10.5 months Median PFS = 4.5 months	N/A	
Takahashi et al. (50)	N/A	29/260 (11.2%)	MST = 13,8 months The survival rates from the first vaccination were 53.5% after 1 year, 36.1% after 2 years, and 8.8% after 5 years.	N/A	185 (71.4%) patients received DC vaccines combined with simultaneous chemotherapy. Patients with an adenocarcinoma had a significantly better prognosis compared to other subtypes (MST 15.3 months vs. 8.8 months; p = 0.003). An erythema reaction at the injection site that was ≥ 30 mm in diameter was correlated most strongly with OS from the first vaccine (MST 20.4 vs. 8.8 months; p<0.001).
Li et al. (51)	No serious AEs Grade 1 temporary exanthema in 1 patient Grade 1 pruritus in 3 patients Grade 1 chills in 1 patient Grade 1 fever in 1 patient Grade 1 fatigue in 1 patient	0/16 (0%)	Survival rates from DC therapy was mentioned in three patients and ranged from 6 to 12 months.	A positive response to DTH skin test was seen in all patients (100%). There was a significant increase in INF-γ expression on day 60 vs. day 0. There was an increasing trend in the mean CD4:CD8 values between day 30 and day 90.	5 patients had disease recurrence or progression of which 3 patients died of stage IV NSCLC.
Lee et al. (52)	Grade 1 flu-like symptoms in 1 patient Grade 1 hemoptysis after each injection in 1 patient Grade 1 nausea in 1 patient Grade 1 fatigue in 1 patient	N/A	MST = 3.9 months	A systemic response against TAA’s was seen in 6/16 patients (37.5%). Tumor CD8+T cell infiltration was induced in 54% of subjects. Patients with increased CD8+T cells following vaccination showed significantly increased PD-L1 mRNA expression.	SD was seen in 25% of patients at day 56
Teramoto et al. (53)	Fever in 16 patients Local reaction at the site in 6 patients Acute lung injury in 1 patient	0/28 (0%)	MST = 7.4 months	MUC1-specific cytotoxic immune responses were seen in 7/7 patients (100%).	A higher MST was seen in patients receiving > 6 vaccinations, in patients with adverse events, and in patients with higher percentage of peripheral lymphocytes. The DCR in the group of patients that had received 6 vaccinations was 42.9%.
Ge et al. (54)	No serious AEs Grade 1–2 fever in 6 patients Grade 1–2 fatigue in 5 patients Grade 1–2 myalgia in 6 patients Grade 1–2 chills in 1 patient	N/A	N/A	A significant decrease in CD3+ CD4+ CD25+ Foxp3+ T regulatory cell number and increase in TNF-α and IL-6 were seen in 2/15 patients (13.3%).	Two patients showed 15% and 64% decrease in CEA and CYFRA21 respectively. The vaccination with the maximum dose significantly improved QOL when administered at the highest dose. In the low dose group: 1 patient had no recurrence, 1 patient had PD and 1 patient had died In de middle dose group: all 3 patients had no recurrence. In the high dose group: 1 patient had died, 1 patient had PD and 7 patients had no recurrence.
***DC/CIK therapy in NSCLC***					
Li et al. (55)	No grade 3 or 4 AEs	N/A	The 2-year OS was significantly increased in the immune-CT group comparing to the non-immunotherapy group (p<0.05).	An increased production of cytokines that have known anti-tumor effects was seen, including IFN-γ, TNF-α and TNF-β, in patients who had no progression, but they were not found in patients who developed recurrence.	The 2-year DFS in the immune-CT group (75.6 ± 7.2%) was higher than that in the CT-group (65.3 ± 8.0%) but there was no significant difference. Immunotherapy was started 1 month after chemotherapy in the chemoimmunotherapy group.
Zhong et al. (56)	Grade 1–3 skin toxicity in 9 patients Grade 1–4 fever in 10 patients	N/A	There was no statistically difference in OS between the chemoimmunotherapy group and chemotherapy group (p = 0.18).	N/A	In the chemoimmunotherapy group, CEA level decreased in 3 patients, and remained stable in 9 patients. The median time to progression in de chemoimmunotherapy group was 6.9 months (95% CI: 5.0-8.8) and 5.2 months (95% CI: 3.3-6.0) in the chemotherapy group (p = 0.03).
Shi et al. (57)	Fever in 4 patients	1/30 (3.3%)	The PFS was significantly increased in the DC/CIK cell group compared to the control group (3.2 vs. 2.56 months; p<0.05).	After treatment, an increase in NK-cells, CD3+ and CD4+ T cells was seen, and a decrease in CD8+ cells.	
Yang et al. (58)	No serious AEs	11/61 (18%)	The 1- and 2-year OS rates were 57.2 and 27% in the chemoimmunotherapy group and were significantly higher than in de chemotherapy group (p<0.05).	A significant increase in the secretion of INF-γ and TNF-α was seen, and a decrease in TGF-β. An enhanced antitumor activity was seen, as well as an increased CD3+CD56+ cell ratio.	There was no significant difference in the survival rate between the adenocarcinoma and squamous carcinoma patients.
Shi et al. (72)	Fever in 3 patients Rash in 14 patients Diarrhea in 9 patients	N/A	The PFS was significantly longer in the DC/CIK plus erlotinib group compared to the erlotinib group (5.02 vs. 3.98 months; p<0.05). There was no statistically significant difference in median OS between both groups (p = 0.29).	An increase in the levels of CD3+, CD4+ and CD8+ T cells was found after therapy in the DC/CIK plus erlotinib group, but not in the erlotinib group.	
Zhao et al. (60)	N/A	N/A	N/A	The serum concentrations of the Th2 cytokines (IL-4 and IL-10) in tumor-bearing patients were significantly higher than those with resected NSCLC before immunotherapy. The post-therapy Th1 cytokine (INF-γ) level in patients with resected NSCLC significantly increased from the pre-therapy level. In tumor-bearing patients, significantly enhanced post-therapy Th2 cytokine (IL-4 and IL-10) levels were found.	
Zhu et al. (61)	Grade 1-2 fever in 5 patients	25/30 (83.5%)	1-year OS was significantly higher in the treatment group than in the control group (83.3% vs. 60.6%; p<0.05).	A significant increase of CD3+, CD4+ and CD4+/CD8+ was seen in the treatment group, but not in the control group.	
Zhang et al. (62)	Grade 1-2 fever in 30 patients Grade 3 fever in 6 patients Grade 1-2 skin rash in 7 patients	N/A	The OS time was significantly increased in comparing to the non-immunotherapy group (p = 0.03).	A positive response to DTH skin test was seen in 59 patients (60.8%).	Matched patients (N = 408) with NSCLC that did not receive DC-CIK acted as the control group.
Zhang et al. (63)	Grade 1-2 fever in 5 patients Grade 1-2 anorexia in 3 patients Grade 1-2 nausea in 3 patients Grade 1-2 radiation pneumonitis in 4 patients Grade 3 radiation pneumonitis in 3 patients	10/21 (47.6%)	The median PFS was significantly longer in the DC/CIK group than in the control group (330 days vs. 233 days; p = 0.0483). There was no difference in median OS between both groups (p = 0.606).	No difference was seen in the serum levels of IL-2 and INF-γ in the two groups both before and after thoracic radiotherapy. No changes were seen in the levels of CD8+ cells or CD4+ cells. A tendency of a decrease in de CD4+/CD8+ T cell ratio was seen in the control group.	
Song et al. (64)	N/A	N/A	N/A	Decreased levels of circulating Tregs and related immunosuppressive cytokines were seen after increased cycles of DC/CIK treatment.	Recurrence rate was lower in the ≥ 3 cycles group compared to < 3 cycles group (p = 0.022).
Chen et al. (65)	Grade 3-4 AEs in 2 patients Grade 1-2 fever in 8 patients Grade 3-4 fever in 1 patients Grade 1-2 chills in 2 patients Grade 3-4 chills in 1 patients	7/31 (22.5%)	OS = 270 days	PD-L1 expression was induced on autologous tumor cells by tumor-reactive DC-CIK cells and elevated IFN-γ secretion was seen.	Among patients with NSCLC, SD was seen in 1 patient.
***AKT-DC therapy in NSCLC***					
Kimura et al. (66)	Fever in 78.0% of the courses Chills in 83.4% of the courses Fatigue in 23.0% of the courses Nausea in 17.0% of the courses	N/A	The 2- and 5-year OS rates were 88.9% and 52.9% respectively.	N/A	The 5-year survival rate of the group that received > 5.0 × 10^10^ cells was better than the group that received less (80.8% vs. 38,5%).
Kimura et al., (67, 68)	Fever in 6.2% of the courses Chills in 6.8% of the courses	N/A	The 2- and 5-year OS rates were 96.0% and 69.4% in group A and 64.7% and 45.1% in group B, respectively, with a HR of 0.474.	The CD8+/CD4+ T cell ratio was higher in the survivors than in the deceased (p = 0.0013).	A higher OS was seen in patients ≤ 55 years (HR 0.0098), male patients (HR 0.474), patients with adenocarcinoma (HR 0.479), patients with stage III cancer (HR 0.399) and patients who did not receive preoperative chemotherapy (HR 0.0483).
***DC therapy in SCLC***					
Chiappori et al. (69)	Grade 1 arthralgia and myalgia in 9 patients Grade 2 arthralgia in 1 patient Grade 1 fatigue in 5 patients Grade 2 fatigue in 1 patient Grade 1 local reaction at the injection site in 5 patients Grade 1 injection site pain in 4 patients	2/54 (3.7%)	The MST from first vaccination was 8.8 months.	A significant p53-specific immune response was seen in 18/43 patients (41.2%).	Patients with a positive immune response had a trend towards improved survival. 78.6% of the patients with a positive immune response had a clinical response to 2^nd^ line chemotherapy compared to 33.3% patients with a negative immune response.
Chiappori et al. (70, 71)	Grade 3 fatigue in 1 patient. Grade 3 toxicities in 8 patients receiving vaccine + ATRA	1/45 (2.2%)	Survival from date of enrollment was numerically higher in arm A (12.2 months), than in arm B (6.3) and arm C (6.2) but not statistically different.	Positive immune responses were obtained in 20% of arm B (vaccine alone) and 43.3% of arm C (vaccine + ATRA).	No difference was seen in OS between patients with a positive immune response and those with a negative immune response (9.2 vs. 9.3 months; p = 0.250).

*Only responses in lung cancer patients are mentioned.

AE, adverse event; AKT, activated killer T cells; ATRA, all-trans-retinoid acid; CEA, carcinoembryonic antigen; CI, confidence interval; CIK, cytokine induced killer; CRC, colorectal cancer; CT, chemotherapy; CTL, cytotoxic T-lymphocyte; DCR, disease control rate; DFS, disease free survival; DTH, delayed-type hypersensitivity; ECOG-PS, Eastern Cooperative Oncology Group-Performance Status; HR, hazard ratio; IL, interleukin; INF-α, interferon-alpha; INF-γ, interferon-gamma; MST, median survival time; N/A, not available; ORR, objective response rate; OS, overall survival; PD, progression of disease; QOL, quality of life; SD, stable disease; TAA, tumor-associated antigen; TNF-α, tumor necrosis factor-alpha; TGF-β, transforming growth factor-beta.

### DC Therapy in NSCLC

The earliest study in this field was performed by Fong and coworkers in patients with metastatic or recurrent cancer who had abnormal or rising serum carcinoembryonic antigen (CEA) levels (35). CEA is a 180-kDa membrane intercellular adhesion glycoprotein that is overexpressed in several malignancies including NSCLC. Twelve patients with either colorectal cancer (CRC) or NSCLC underwent peripheral blood leukapheresis after prior administration of Flt3 ligand, a hematopoietic growth factor known to expand DCs *in vivo*. DCs were subsequently harvested and loaded with a nonapeptide derived from a human leucocyte antigen (HLA)-A0201-specific peptide of CEA, as well as with keyhole limpet hemocyanin (KLH), a protein with adjuvant properties that also allows to monitor therapy-induced immune responses. Patients were injected intravenously (i.v.) with progressively increasing doses of antigen-exposed DCs with a maximum of 10^9^ cells. Adverse events (AEs) were mild self-limited rigors and fever (7/12), as well as mild diarrhea (5/12). Vaccination elicited a CEA-specific immune response in seven patients. Two out of twelve patients experienced dramatic tumor regression, one patient had a mixed response, and two had stable disease (SD). Clinical responses correlated significantly with the expansion of CD8+ T cells.

A similar CEA-targeted DC vaccination strategy was used by the group of Itoh and Ueda et al. (36, 41). The first study enrolled ten patients with advanced digestive tract or lung cancer expressing CEA (36). PBMCs were harvested from peripheral blood by leukapheresis after five days of priming with granulocyte colony-stimulating factor (G-CSF) and cultured with granulocyte/macrophage colony-stimulating factor (GM-CSF) and interleukin 4 (IL-4) to generate DCs. The DCs showed an immature phenotype and were loaded with CEA652, a nonapeptide restricted to HLA-A24, which is present in 60% of the Japanese population. Patients received repeated intradermal (i.d.) and subcutaneous (s.c.) injections up to a cumulative dose ranging from 2.7 × 10^7^ to 1.6 × 10^8^ DCs. Seven patients also received adjuvant interferon-alpha (IFN-α) and tumor necrosis factor-alpha (TNF-α) twice a week during the vaccination period. No severe toxicity was observed. A positive response to delayed-type hypersensitivity (DTH) skin test was seen in two patients. One of the two demonstrated also a CEA-specific immune response. Two patients, of which one with stage IV lung cancer, had SD for 6 and 9 months respectively, associated with a continuous decrease of serum CEA in the first patient. Clinical and immunological responses were only observed in patients treated with adjuvant use of IFN-α and TNF-α so it is not known whether these responses could be attributed to the DC vaccine. In a follow-up study, 18 patients (five patients with lung cancer) were enrolled and treated using the same immunization protocol, without cytokine adjuvants (41). The vaccine was well tolerated and no toxicity was observed. Although no tumor shrinkage occurred in any patient, long-term SD or marked decreases in the serum CEA level were observed in some subjects. A positive skin response to CEA652-pulsed DCs and a positive *in vitro* cytotoxic T-lymphocyte (CTL) response to CEA652 peptide after therapy were seen in most of the patients in whom treatment was clinically effective.

Other studies also selected CEA as the antigen of choice for active immunotherapy with DCs. A phase 1 trial included one patient with metastatic lung adenocarcinoma who underwent four monthly immunizations with autologous DCs transfected with CEA-encoding RNA and total tumor RNA (37). Both CEA-specific and tumor-specific CTL immune responses were seen, of which the latter were greater. The authors conclude that RNA-transfected DCs can induce antigen-specific T cell responses in cancer patients with surgically resected malignancies. Morse and coworkers investigated the safety and clinical and immunological efficacy of a DC vaccine modified with a recombinant Fowlpox vector encoding CEA and a triad of stimulatory molecules [rF-CEA(6D)-TRICOM], injected both i.d. and s.c. (43). 14 patients with metastatic CEA-expressing malignancies were enrolled, of which three with NSCLC. There were no treatment-related grade 3 or 4 AEs. One patient had a significant decrease in the CEA level and a minor regression in a retroperitoneal and supraclavicular adenopathy. Five other patients were stable through at least one cycle of immunization. A CEA-specific immune response among both CD4+ and CD8+ T cells was seen in all evaluable patients. There was a trend towards a greater peak frequency of CEA-specific T cells among those with either a minor response or a SD.

Kontani et al. evaluated the clinical effects of a DC vaccine targeting the TAA mucin 1 (MUC1) in 14 patients with advanced or metastatic breast or lung cancer (38). MUC1 is a glycoprotein that is markedly hypoglycosylated in cancer compared to normal tissues, leading to the exposure of immunogenic epitopes (73). PBMCs were collected from peripheral blood samples and supplemented with IL-4 and GM-CSF. Subsequently, DCs were loaded with MUC1 peptides or tumor lysate obtained from malignant pleural effusion specimens of the patients. DCs were then injected s.c. in the supraclavicular region or intrapleurally, at least three times at 2-week intervals. Fever occurred in seven patients. After vaccination, all the evaluable patients with MUC1-positive cancer acquired antigen-specific immunity compared to only one patient with MUC1-negative cancer. Reductions in tumor sizes or tumor marker levels or disappearance of malignant pleural effusion were seen in seven of nine MUC1-positive cancers. The survival of MUC1-positive patients was significantly longer compared to MUC1-negative patients (16.8 vs. 3.8 months; p = 0.0101). The authors conclude in this study that this tumor antigen can elicit a strong immune response and that DC vaccines targeting MUC1, which is expressed in 60% of the lung cancer patients, are a promising immunotherapy in the treatment of cancer (38). Of note, similar signals of clinical efficacy were observed with other MUC1-targeted vaccine approaches in NSCLC (20, 74).

Some important conclusions can be drawn from the different studies mentioned above. First, DC therapy containing one TAA is well tolerated with only minor side effects observed. For CEA-targeted DC vaccination studies, this is reassuring given the severe pulmonary toxicity observed with CEA-specific CAR T cell therapy, which is related to the expression of this antigen on normal pulmonary epithelium (75). Second, this vaccination approach appears to elicit an antigen-specific, T cell-mediated immune response in a substantial fraction of lung cancer patients, despite a number of obstacles: 1) the use of a shared TAA for which high levels of immunological tolerance must be overcome, 2) the injection of immature DCs in some trials, and 3) the climate of systemic immune suppression in the advanced cancer patients enrolled. Yet, clinical responses were rare, possibly explained by the fact that only one tumor antigen was targeted. A limitation of peptide-based formulations is that they are HLA-restricted, which necessitates patient selection. This is not the case for other antigen formulations such as mRNA encoding antigens or tumor lysates that were used in some other trials. A remarkable observation from these studies is also that prior mobilization with Flt3 ligand or G-CSF could expand the number of DCs produced. However, because it was not assessed whether higher DC doses also yielded stronger immune responses, the benefit of mobilization of the donor in the DC manufacturing process remains unclear.

In contrast to the aforementioned trials enrolling different tumor types, the first DC vaccination trial exclusively in NSCLC patients was performed by Hirschowitz et al. (39, 40). In this trial, multiple TAAs were targeted simultaneously. Autologous DC vaccines were delivered to 16 individuals with stage IA to IIIB NSCLC treated with surgery, chemoradiation, or multimodality therapy. DCs were generated from CD14+ precursors and pulsed with apoptotic bodies of an allogeneic NSCLC cell line that overexpressed human epidermal growth factor receptor 2 (HER2/neu), CEA, wilms tumor protein 1 (WT1), and survivin. Interestingly, DCs were only “partially” matured. Patients received two i.d. vaccines with 1 month apart (average dose of 9.1 × 10^7^ and 8.2 × 10^7^ cells per immunization respectively). AEs were limited to a mild skin reaction at the injection site (10/16) and minor fatigue for one to two days after injection (3/16). Of the 16 patients, six showed an antigen-specific response and five showed a tumor-antigen independent response. Five individuals had documented disease recurrence or progression of which three succumbed to the disease. One individual with stage IB NSCLC developed a solitary brain metastasis 2 months following the initial vaccine and had no evidence of disease 15 months following metastasectomy. Two patients with unresectable stage III NSCLC showed no signs of disease progression at 35 and 23 months from chemoradiation, respectively. The aforementioned clinical outcomes were independent of measured immunologic responses. The same group conducted a continuation study with similar inclusion criteria and immunization protocol, using an immature DC vaccine (44). 14 patients were enrolled of which seven had undergone surgical resection, with or without adjuvant therapy, and seven with unresectable stage III who had been treated with chemoradiation. Immunologic responses, measured by IFN-γ enzyme-linked immune absorbent spot (ELISPOT), were seen in 3/7 stage III unresectable, and 6/7 stage I/II surgically resected patients. There were no AEs, except for local reactions in most subjects. The authors conclude that immature DCs pulsed with apoptotic tumor cells have similar biologic activity to a matured DC preparation in a similar clinical protocol (44).

Although clinical outcomes were difficult to interpret, probably due to the heterogeneity of the patient population, and not correlating with immunological responses, the studies of Hirschowitz and colleagues have clearly indicated that DC therapy following surgery, chemoradiation or multimodality treatment is safe and can possibly find its role as an adjuvant treatment. A remarkable observation from these clinical trials was that immature DCs were able to elicit immune responses in almost 2/3 of the patients, since it has been appreciated for a long time that these DC subsets rather induce immune tolerance than immune stimulation. A difference with the previous studies is that an allogeneic tumor cell line was used to produce a multivalent vaccine, targeting several TAAs. Yet, the antigenic make-up of the cell line used may not be representative for each patient’s tumor.

Further exploiting the idea of targeting multiple antigens, Perroud and coworkers assessed the feasibility, safety and immunologic response of a mature, antigen-pulsed autologous DC vaccine loaded with peptides of WT1, CEA, HER2, and Melanoma Antigen 1 (MAGE-1). The trial enrolled five patients with inoperable stage IIIB and IV NSCLC (47). All patients received prior conventional treatment (chemotherapy with or without radiotherapy). PBMCs, obtained after leukapheresis, were cultured in a medium with GM-CSF and IL-4, and subsequently activated with IFN-γ. Patients received two doses of 5 × 10^7^ DCs administered s.c. and i.v. two times at 2-week intervals. One patient developed grade 2 fatigue and chills following the first dose of the vaccine. A lymphoproliferation assay showed an improvement in the specific immune response after immunization in all patients, with a tendency to wane after the second vaccine dose. Survival from the last dose of the vaccine ranged between 82 and 277 days. Three patients had a longer survival time than expected for their tumor, node and metastasis (TNM) classification. The fact that immune responses were not long lasting possibly indicates that multiple doses of the vaccine are required to achieve clinical efficacy.

Li et al. reported the results of a phase 1 trial enrolling 16 patients with stage I to IIIB NSCLC (51). All had no evidence of progression at the time of enrollment and had completed definitive therapy (surgical, medical or multimodal). DC immunotherapy was generated from the patient’s PBMCs and loaded with recombinant survivin and MAGE-3 peptides. To induce DC maturation, a cytokine cocktail consisting of IL-1β, IL-6, TNF-α, IFN-γ, prostaglandin E2 (PGE2), and poly I:C had been added to the culture. A prime immunotherapy (9.1 × 10^7^ cells/dose) and a single boost (8.2 × 10^7^ cells/dose) were administered i.d. 1 month apart. AEs were grade 1 fever, chills and fatigue in one patient, and grade 1 pruritus in three patients. A positive response to DTH skin test was seen in all patients. There was a significant increase in IFN-γ expression on day 60 versus day 0. There was also an increasing trend in the mean CD4:CD8 values between day 30 and day 90; however, the increase was not statistically significant. In total, 5/16 patients experienced disease recurrence or progression, of which three patients succumbed to the disease.

An alternative approach to target multiple antigens simultaneously is to load DCs with autologous tumor cells or cellular lysates. Chang et al. for example conducted a pilot trial using mature DCs pulsed with necrotic tumor cells enriched from malignant pleural effusion specimens (42). Eight patients with advanced NSCLC were injected with antigen-loaded DCs into the inguinal lymph nodes under ultrasound guidance. No major toxicities occurred. Six patients received all six DC injections. Of these, two patients had SD and one patient had a minor response. Minor to moderate increases in T cell responses against tumor antigens were observed after DC vaccination in six of eight patients. The two patients who had a longer disease control also developed better T cell responses. The immunological and clinical effects of a DC vaccine pulsed with autologous tumor lysate was also assessed by the groups of Mayordomo et al. (45) and Um et al. (46). In the first study, 15 patients with metastatic cancer (two with NSCLC) underwent mononuclear cell apheresis after prior mobilization with GM-CSF. PBMCs were cultured with IL-4 and GM-CSF. DCs were then administered i.v. with a median dose of 6.2 × 10^7^ cells per vaccination. In addition, IL-2, IFN-α, and GM-CSF were co-injected s.c. as an adjuvant for several days. A positive response to DTH skin test was noted in 9/15 patients after the first immunization. Seven patients, of whom one with NSCLC, had SD for more than 3 months and seven other patients experienced disease progression. AEs were mild and included fever immediately after DC infusion in six patients and asthenia in eleven patients. The second study enrolled exclusively subjects with stage IIIB and IV NSCLC. DCs were again loaded with autologous tumor lysate by a combination of electroporation and passive loading. Autologous tumor samples were obtained from bronchoscopic biopsies, surgical samples or lymph node biopsies. The antigen-loaded immature DCs were subsequently activated with TNF-α, IL-1, IL-6, and PGE2. In this phase 1 dose-escalation study, 15 patients were assigned to cohorts that received 3, 6, or 12 × 10^6^ DCs by i.d. injection. The maximum dose of the vaccine was shown to be safe with only one patient experiencing low grade fever. In 5/9 patients, the vaccine resulted in an increased IFN-γ production by peripheral blood CD8+ T cells. However, a relationship between the immunological response and the vaccination dose was not seen. Clinical responses were assessed in eight patients. All had PD. Nevertheless, there were mixed responses that fulfilled PD definition but demonstrated some clinical benefit in two patients.

Again, clinical outcomes were disappointing. A possible explanation is that most of the patients enrolled in these studies suffered from relapsed or refractory cancer with often bulky disease and a worse performance status, which is shown to be less responsive to DC vaccination. Another potential concern may be the high concentration of suppressive factors released from the tumor cells, which may influence DC functionality. Moreover, in the last study, the autologous tumor samples used for making tumor lysate had been obtained before the initiation of chemotherapy. Changes in the tumor antigenicity during treatment could perhaps explain the low clinical efficacy.

In an attempt to circumvent the limitations of typically small sample sizes in DC vaccination trials, the group of Takahashi and coworkers conducted a pooled retrospective analysis of 62 patients from one center. The patients had previously treated inoperable or postoperatively relapsed NSCLC and received activated DCs pulsed with either autologous tumor lysates or peptide antigens (WT1, MUC1, CEA) matched to their HLA-A type (48). The DCs were activated by *in vitro* exposure to OK-432, which is a clinically approved lyophilized mixture of group A Streptococcus pyogenes known to promote functional maturation of DCs, including the capacity to secrete IL-12. The vaccines were injected i.d. near the axillar and/or inguinal lymph nodes with a median of 10 immunizations (range, 4–31). Clinical responses were observed in five patients, of which one patient with a complete response (CR). Another 26 patients developed SD. Median survival time (MST) was 12 months from the first DC vaccination. Of note, standard chemotherapy was continued in 36 patients during DC vaccination. A better OS was found in patients who received more than five vaccinations and those with the best performance status. Multivariate analyses also revealed that the use of WT1 peptides significantly affected OS both from initial diagnosis and from the first vaccination. Furthermore, no serious AEs related to the vaccine were observed. In an extended analysis, 260 patients with locally advanced or metastatic NSCLC at six centers were analyzed (50). All had received five or more WT1 and/or MUC1 peptide-pulsed DC vaccinations once every 2 weeks. In some patients, OK-432 was co-administered i.d. as an immunological adjuvant simultaneously with the vaccine. In the majority of the patients (71.4%), DC vaccination was combined with chemotherapy. MST from first vaccination was 13.8 months (95% CI 11.4–16.8) with 8.8% being alive after five years. Patients with an adenocarcinoma had a significantly better prognosis compared with other subtypes (MST 15.3 vs. 8.8 months; p = 0.003). An erythema reaction at the injection site that was ≥ 30 mm in diameter was strongly correlated with OS from the first vaccine (MST 20.4 vs. 8.8 months; p<0.001). Another Japanese group retrospectively analyzed data of 40 patients with MUC1-positive NSCLC treated with a MUC1-targeted and OK-432 activated DC-vaccine, exploring predictive biomarkers for clinical responses. All patients had stage IIIB-IV NSCLC that was refractory to standard anticancer therapies (53). The vaccines were injected s.c. in the axilla or supraclavicular fossa every 2 weeks until disease progression. Low-grade fever occurred in 16 patients and local skin reactions in six individuals. No patients achieved an objective response. The MST after initial vaccination was 7.4 months and the 1-year OS was 25%. Patients who received more than six vaccinations had a longer MST and 1-year OS (9.5 months and 39.3% respectively). Interestingly, in the latter group, patients who developed immune-related AEs had a significantly longer MST compared with patients without those AEs (12.6 vs. 6.7 months; p = 0.042). In addition, longer survival was also seen in patients with > 20% lymphocytes prior to vaccination (12.6 vs. 4.5 months; p = 0.014). All seven patients who had received six vaccinations and were evaluable for immune responses showed an increase in MUC1-specific T cells and a decrease in Tregs.

A major drawback of these studies is however their retrospective design, limiting the interpretation of the results. Since most of the patients also received simultaneous chemotherapy in the first two studies, and no control group was applied, it is difficult to draw definite conclusions regarding the clinical benefit of the vaccine. Furthermore, it was appreciated that patients receiving more DC vaccines also had better survival outcomes, which is of course interesting since the optimal DC dose and frequency of administration is not yet determined. However, this survival benefit could have been possibly attributed to the better performance status of the patients in the group treated with the highest cumulative dose.

Knowledge of negative feedback pathways controlling inflammatory responses can be exploited to re-engineer DCs. Based on this concept, Ge et al. evaluated the safety and efficacy of a DC vaccine activated using the Toll-like receptor (TLR) agonist flagellin, together with siRNA-mediated silencing of the gene encoding for suppressor of cytokine signaling 1 (SOCS1) (54). SOCS1 has been shown to be a negative regulator of DC activation and IL-12 production, thus restricting the DC’s capacity to break immunological tolerance. By analogy to other trials, the DCs were pulsed with peptides of survivin and MUC1. Just as MUC1, survivin is also frequently overexpressed in NSCLC and contributes to oncogenesis. In this phase 1 dose-escalation trial, 15 patients with resected stage I to III NSCLC were i.v. injected with 1 × 10^6^, 1 × 10^7^, or the maximum dose of 1 × 10^8^ DCs at days 7, 14, and 21. The most common AEs were grade 1 flu-like symptoms, which occurred mostly in the group immunized with the maximum dose of the vaccine. A significant decrease in T-regulatory (Treg) cells and increase in TNF-α and IL-6 were seen in two patients. Two patients also showed a 15% and 64% decrease in CEA and CYFRA21, respectively. Interestingly, the patients’ quality of life (QOL) was significantly improved in the high-dose group, compared with the low-dose and middle-dose group after treatment. More importantly, in the long-term follow-up after more than four years, only two patients had died, two patients had progressive disease (PD) and 11 patients had still no recurrence. With the use of SOCS1-silencing, this trial is the first in lung cancer to explore targeted genetic re-engineering of DCs to boost immunogenicity. This manipulation did not translate into increased cytokine-mediated toxicity. Still the added value of SOCS1-silencing in terms of clinical outcome cannot be ascertained from this trial as there was no comparator product treated with a control siRNA.

Based on preclinical evidence pointing to a possible synergistic effect between chemotherapy and vaccination (as discussed below), Hu et al. explored the combination of pemetrexed and DCs pulsed with autologous tumor lysate in 27 patients suffering from stage IIIB or IV lung adenocarcinoma who had failed on maintenance gefitinib or erlotinib treatment after platinum-doublet chemotherapy (49). PBMCs were enriched from a 50-ml blood sample using density gradient centrifugation and subsequently cultured in the presence of IL-4 and GM-CSF. DCs were then given i.d. every 3 weeks at day 8 of each chemotherapy cycle. Grade 1 fever after DC therapy was noted in five patients. Other, mostly hematological, toxicities were attributed to chemotherapy. Three patients (11.1%) experienced a partial response (PR). The median progression-free survival (PFS) was 4.5 months and the median OS 10.5 months, which is better than a previous trial with second line pemetrexed in advanced NSCLC (76). Grade 1 fever after DC therapy was noted in five patients. Other, mostly hematological, toxicities were attributed to chemotherapy. This was the first study to prospectively investigate the added value of DC therapy combined with chemotherapy. However, since there was no control group, the real value of DC vaccination in this setting remains to be confirmed.

In contrast to all the trials using systemic injection of DCs, Lee and coworkers explored the intratumoral injection in terms of feasibility, safety and efficacy. In a phase 1 dose escalation study, autologous DCs were administered intratumorally in 16 subjects with stage IIIB and IV NSCLC (52). Interestingly, the DCs were genetically modified by transduction with an adenoviral (Ad) vector expressing the *CCL21* gene (Ad-CCL21-DC). CCL21 is a lymphoid chemokine that strongly attracts effector T cells and DCs and hence facilitates entry into the tumor and *in situ* vaccination. Endpoints were safety and tumor antigen-specific immune responses. Patients enrolled into a given cohort received the same Ad-CCL21-DC dose (1 × 10^6^, 5 × 10^6^, 1 × 10^7^, or 3 × 10^7^ cells/injection) by CT-guided or bronchoscopic intratumoral injection on days 0 and 7. Three patients developed possibly treatment-related AEs (flu-like syndrome, hemoptysis, nausea and fatigue, all grade 1). Twenty-five percent of the patients had SD at day 56. MST was 3.9 months. A systemic response against TAAs was observed in six of 16 patients by means of an IFN-γ ELISPOT assay. Tumor CD8+ T cell infiltration was induced in 7/13 subjects. Interestingly, intratumoral PD-L1 mRNA expression increased significantly with increased CD8+ T cell infiltration following vaccination. The authors of this study suggest that *in situ* vaccination itself increases PD-L1 expression as a result of antigen recognition and CD8+ T cell infiltration at the tumor site. In this way, vaccination may be an effective approach to enhance the efficacy of PD-1/PD-L1 checkpoint inhibitors in “cold” tumors with low PD-L1 expression and/or a lack of CD8+ T cell infiltration (52). Still, the major question remains whether DC-induced T cell infiltration and potential priming at one injected site will induce T cells capable of homing into and controlling other metastatic sites.

### DC/CIK Cell Therapy in NSCLC

In recent years, the use of autologous DCs co-cultured with cytokine-induced killer (CIK) cells has been the subject of numerous trials in NSCLC, all of them conducted in the Far-East region (77). CIK cells are a subset of non-MHC restricted natural killer T-lymphocytes with a CD3+ CD56+ phenotype that can proliferate rapidly *in vitro* and display strong cytolytic activities against malignant cells (59). In DC/CIK therapy, the DCs are derived from mononuclear cells obtained by leukapheresis in typical GM-CSF/IL-4-supplemented medium and loaded with antigens (autologous tumor lysate or peptides). CIK cells are generated by culturing PBMCs in medium supplemented with anti-CD3 antibody, recombinant human IL-1a, IFN-γ, and IL-2 (58).

DC/CIK cell therapy has been evaluated in diverse disease settings: as adjuvant therapy combined with chemotherapy in resectable disease, in stage IIIB and IV patients as first line in combination with chemotherapy, and as a maintenance treatment after first line chemotherapy (55–58, 60, 62, 64, 72). In total, 646 patients were enrolled in these trials. No serious toxicities were observed. Signals of clinical activity were observed in some cases, albeit usually modest and often without statistical significance. Signs of systemic immune activation were reported in patients receiving the experimental arm, including increased numbers of circulating CD8+ and CD4+ T cells, a shift from a Th2 toward a Th1-polarized immune response profile with an increase of the anti-tumoral cytokines IFN-γ, TNF-α, and TNF-β (albeit only in early-stage patients), and a reduction in Tregs after repeated injections of DC/CIK.

A different concept is the combination of DC/CIK cell therapy with thoracic radiotherapy (TRT) or chemoradiotherapy (CRT). The underlying rationale being that radiation-killed tumor cells release tumor antigens and “danger-associated molecular patterns” that can potentially promote DCs to elicit tumor antigen-specific CD8+ T cell responses, which would further consolidate or amplify objective responses and improve survival outcomes (61, 63). The immunogenic effects of radiotherapy are thought to underlie the positive results of MUC1-targeted vaccination and, more convincingly, adjuvant PD-L1 blockade in stage III NSCLC patients treated with chemoradiation (20, 78). In a phase 2 trial, patients with stage III and IV NSCLC received TRT (60 Gy delivered at 2 Gy per fraction) plus MUC1-loaded DC/CIK cell therapy or TRT alone (63). All subjects had previously been treated with two or more cycles of platinum-based doublet chemotherapy without disease progression. Patients that received DC/CIK cells combined with TRT had a longer PFS than those who received TRT alone (330 days vs. 233 days; p<0.05), as well as a better ORR (47.6% vs. 24.6%; p<0.05). Median OS was not significantly different between the two groups. Zhu et al. conducted a RCT in 63 patients with stage IIIB NSCLC (61). Of these, 30 patients were treated with DC/CIK cell therapy combined with platinum-based doublet CRT. DCs were not loaded with tumor antigens. The ORR was significantly higher in the group treated with DC/CIK and CRT than in the group treated with CRT alone (83.3% vs. 54.5%; p = 0.014). One-year survival rate was also better (83.3% vs. 60.6%; p<0.05). These studies suggest that combined treatments with DC/CIK cell therapy and (chemo)radiotherapy can enhance tumor responses and prolong survival.

Recently, an interesting variation on the DC/CIK manufacturing process was reported and evaluated in advanced cancer patients, among them five with NSCLC. In a phase 1 trial by Chen et al., DC/CIK cells were further activated *in vitro* by incubation with the anti-PD-1 antibody pembrolizumab, and administered i.v. by repeatedly infusions (65). Patients were progressive after at least one previous course of appropriate anti-tumoral treatment. Of note, grade 3 or 4 treatment-related AEs (fever, chills) were noted in two patients. ORR was 22.5% with a median OS and PFS of 270 and 162 days respectively. Still, the actual added value of *in vitro* activation with anti-PD-1 is not clear from this trial as there was no comparator arm with “standard” DC/CIK infusions.

### AKT-DC Therapy in NSCLC

Another form of adoptive immunotherapy involving DCs, although somewhat different from the aforementioned treatments, is a therapy using autologous activated killer T cells and DCs (AKT-DC) obtained from tissue cultures of the tumor-draining lymph nodes of the primary lung tumor. Kimura et al. demonstrated that the tumor-draining lymph nodes of lung cancer patients are a potent source of killer T cells specific to autologous tumor cells, but also of mature DCs, when cultured with low dose IL-2, and that this *in vitro* expansion of T cells could last for up to 2 months (79). Based on this mechanism, a phase 2 trial was conducted evaluating the safety and feasibility of chemo-immunotherapy using these AKT-DCs in post-surgical N2 NSCLC patients (66). 31 patients were enrolled, of which three subjects eventually dropped out. Four courses of chemotherapy were administered along with AKT-DC immunotherapy every 2 months for 2 years. Fever and chills were the most frequent AEs. The 2- and 5-year OS were 88.9% and 52.9%, respectively.

The same group performed a phase 3 RCT investigating the efficacy of adjuvant chemo-immunotherapy with AKT-DC, targeting residual micrometastases, in 103 patients with resected NSCLC (67, 68). Patients were randomly allocated to receive either chemo-immunotherapy (group A) or chemotherapy alone (group B). Those who were assigned to group A received four courses of platinum-based chemotherapy along with AKT-DC immunotherapy for up to two years after surgery. Almost half of the patients treated with immunotherapy had at least one AE, mostly chills and/or fever. The 2- and 5-year OS rates were 96.0% and 69.4% in group A and 64.7% and 45.1% in group B, respectively, with a hazard ratio (HR) of 0.474. Subgroup analysis also showed that younger patients, male patients, patients with adenocarcinoma, patients with stage III cancer and those who did not receive preoperative chemotherapy had a significantly better OS. This study showed that NSCLC patients could benefit from adoptive cellular immunotherapy as an adjuvant to surgery. However, the heterogeneity of the study population was a major limitation.

### DC Therapy in SCLC

The stark differences in biological and clinical behavior of SCLC compared to NSCLC are also reflected at the immunological level. As a demonstration, clinical trials to this date show only limited responses to immune checkpoint inhibition in this aggressive tumor, in contrast to NSCLC (80, 81). Also, DC-based immunotherapy trials in SCLC are scarce. Antonia and Chiappori were the first to test the immunological and clinical effects of a cancer vaccine consisting of DCs transduced with an adenovirus expressing p53 (Ad.p53) in patients with extensive disease SCLC (69, 82). The tumor suppressor gene, p53, plays an important role as a regulator of cell growth and differentiation and is mutated in ≥ 90% of the SCLC cases (82). Hence, it is considered as an ideal TAA. Fifty-four patients were enrolled in this phase 1/2 trial. All patients were treated with conventional chemotherapy prior to the immunizations. PBMCs were obtained after leukapheresis and cultured in media supplemented with GM-CSF and IL-4. At the completion of incubation, DCs were subsequently infected with Ad.p53 at a viral particle to cell ratio of 15,000:1. DCs had a mature phenotype. Patients were scheduled to receive three doses of the vaccine i.d. at 2-week intervals. Those who did not progress after three immunizations underwent a second leukapheresis and received three additional doses of the vaccine, but this time once a month. The number of injected DCs was limited to 5 × 10^6^ cells. p53-specific T cell responses were observed in 18/43 (41.8%) patients by IFN-γ ELISPOT assays. AEs associated with the vaccine were infrequent and mostly mild, with one patient experiencing grade 2 fatigue and one patient grade 2 arthralgia. Two patients achieved a PR and 13 patients had SD. Remarkably, a high rate of ORRs to second line chemotherapy was seen in patients with a positive immune response (78.6%) compared to patients with a negative immune response (33.3%). This is higher than expected based on previous trials with paclitaxel in patients with extensive SCLC (83, 84). Median OS was 8.8 months from the time of first vaccination. Patients with a positive immune response to vaccination had a trend towards an improved survival (MST 12.6 vs. 8.2 months; p = 0.131).

The same group subsequently conducted a randomized phase 2 trial involving 69 patients with extensive SCLC who were responsive to therapy or had non-progressive disease after first-line conventional chemotherapy (70, 71). Subjects were randomized into three arms: arm A (control group), arm B (Ad.p53-DC vaccine only), or arm C (Ad.p53-DC vaccine plus All-trans retinoic acid (ATRA)). The rationale to use ATRA is that it decreases systemic levels of myeloid-derived suppressor cells (MDSCs), which have potent immunosuppressive activity. The same immunization protocol was applied as the previous study. The vaccine was found to be safe with one patient experiencing grade 3 fatigue in arm B and eight patients experiencing grade 3 toxicities in arm C. Positive immune responses were obtained in 3/15 of the patients in arm B and 10/23 patients in arm C. The ORRs to second-line chemotherapy were 15.4%, 16.7%, and 23.8%, respectively for arms A, B and C with no survival differences between the different arms. These ORRs were lower than in the previous studies with the same vaccine. Surprisingly, survival from date of enrollment was numerically higher in the control arm than in de treatment arms (12.2, 6.3, and 6.2 months, respectively). A major limitation of this study was the high dropout rate which prevented patients from completing at least one cycle of salvage chemotherapy. Despite this limitation, some conclusions can also be drawn. First, the safety of the Ad.p53-DC vaccine was confirmed and second, the vaccine was able to elicit a specific cytotoxic T cell response in 20-40% of the patients with extensive SCLC, possibly influenced by the co-administration of ATRA. However, this did not translate into clinical responses, which were poor. The higher-than-expected response rate to second line paclitaxel in the first trial is encouraging and paves the way to combinatorial approaches of chemotherapy with immunotherapy to improve clinical efficacy.

## Discussion

For almost 20 years, long before the introduction of checkpoint inhibitors, DCs have been studied as a form of immunotherapy in lung cancer patients. This was based on a large body of preclinical data demonstrating the power of DCs to elicit *de novo* cytotoxic T cell responses, and the presence of different classes of TAAs in lung cancer. Evidence, mostly from phase 1 clinical trials, indicates that DC-based immunotherapy is safe and well tolerated with minor side effects depending on the route of administration. Local reactions (e.g., erythema) are a commonly reported AE after cutaneous injection, while systemic side-effects such as fever, chills and fatigue can be triggered as well. These AEs are mostly mild and transient. Severe toxicities rarely occur when DC-based immunotherapy is given solely. This is in contrast to the sometimes serious AEs seen with checkpoint inhibitors. Moreover, even in DC therapies incorporating whole tumor preparations, hence containing a substantial fraction of self-antigens, no clinically significant signs of auto-immunity have been reported so far.

Active immunotherapy involving DCs aims at eliciting cellular immunity in a tumor-specific and robust way. Data from the aforementioned early-phase trials demonstrate that antigen-specific immune responses can be observed in a significant number of patients, even in individuals with metastatic disease. However, positive immune responses as measured by a DTH skin test correlate only imperfectly with clinical outcomes, as shown in other tumor types (85, 86). In addition, these immunological responses tend to abate after the last injected dose.

Despite their proven immunogenicity, DC-based immunotherapy delivers low response rates, with 9.3% (40/432) of the lung cancer patients achieving an objective response. This is comparable to the ORR of second-line docetaxel in metastatic NSCLC (albeit with much less toxicity) and is lower than second-line PD-1/PD-L1 immunotherapy in the same, unselected population (8–11). In SCLC, traditionally considered as a “cold” tumor, the ORR is even lower (3.0%). However, higher ORRs were obtained when DC-based vaccination is combined with CIK cell therapy and/or concurrent chemotherapy (31.2%). Since most of the trials were not designed to assess OS, survival data of DC vaccination in lung cancer patients are scarce and anecdotal. Moreover, a remarkable observation in the DC vaccination field is the disconnect between clinical response and survival, as seen with sipuleucel-T, the FDA-approved DC vaccine for castration-resistant prostate cancer.

**Figure 2:**
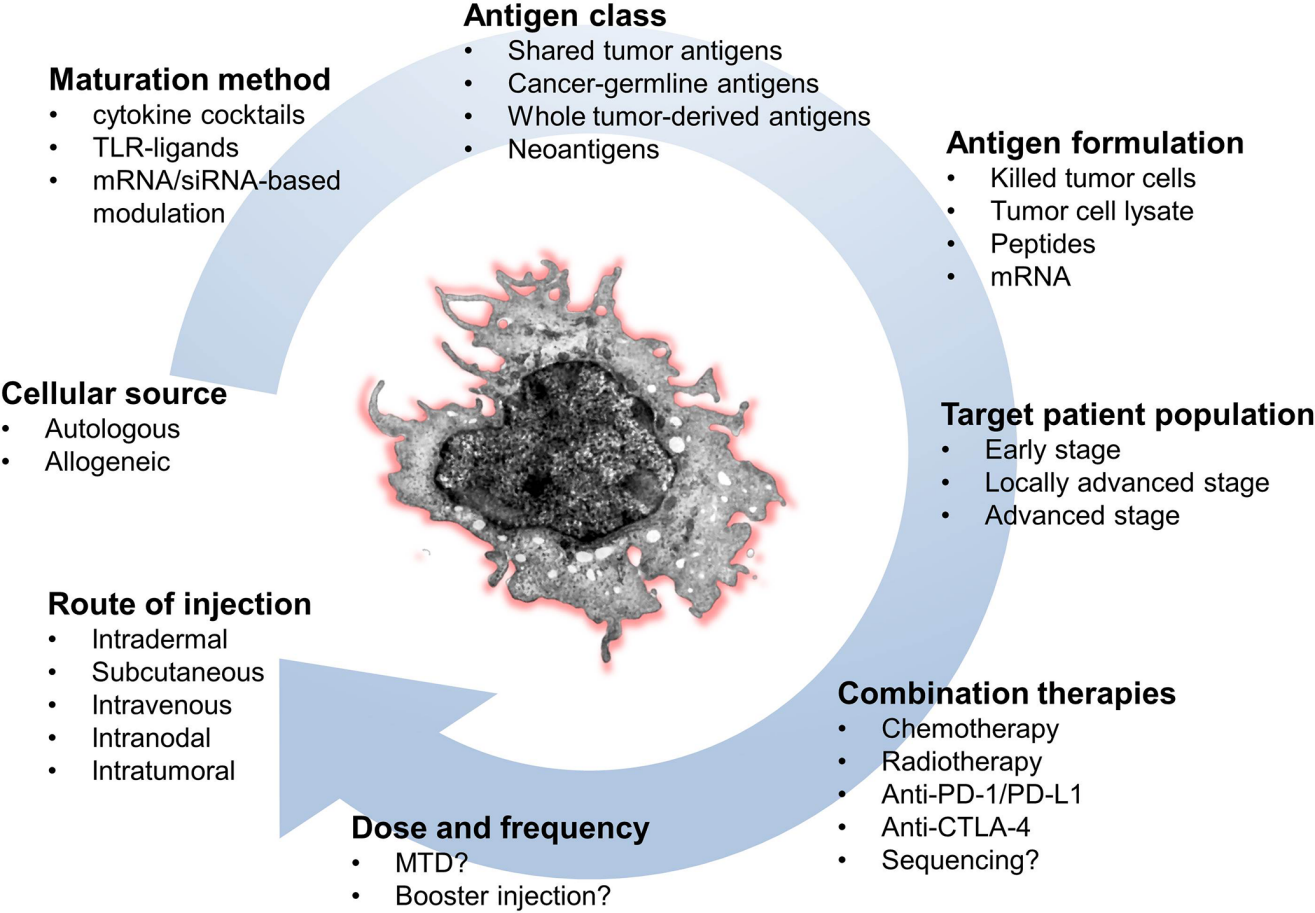
Key parameters to optimize the success of DC-based immunotherapy. CTLA-4, cytotoxic T lymphocyte-associated antigen 4; DC, dendritic cell; MTD, maximum tolerated dose; PD-1, programmed cell death protein 1; PD-L1, programmed death-ligand 1.

A typical limitation of the published studies is the small number of patients and the lack of a control group in almost all clinical trials. Another complicating factor is the huge variability in the methods used. This comprises differences in the type and formulation of TAAs, the DC maturation state at the time of vaccination, different use of co-delivered immunostimulants, as well as variations in the route and frequency of DC injection and dose of the vaccine. Ideally, each of these parameters needs to be optimized in order to improve the clinical efficacy of DC therapy (**Figure 2**).

Different antigen formulations have been used in DC therapy for lung cancer, with tumor-derived peptides (single or combination) and undefined antigen preparations such as autologous tumor cells or cellular lysates being the most frequently used sources. While the use of peptides imposes restrictions in terms of the HLA-type of the target patient population, this is not the case for antigen preparations which also have the advantage to potentially target a much broader antigenic repertoire corresponding more closely to the patient’s tumor. A limitation in this approach is the often limited access to autologous tumor material for antigen extraction, as is the case in metastatic lung cancer. Furthermore, in some clinical trials, immature DCs were used in the vaccination protocol. While immunological responses were occasionally observed, immature DCs are primarily inducers of immunological tolerance, which is obviously unwanted in the setting of cancer immunotherapy. To achieve maturation, DCs can be exposed to a myriad of molecular combinations. However not all of them can be easily implemented in a clinical-grade production process, either due to stability, toxicity concerns and/or cost. In addition, strong stimuli can lead to the phenomenon of DC “exhaustion” whereby the capacity to produce the type-1 polarizing cytokine IL-12 is lost by the time the cells reach T cells *in vivo*. Also, inflammatory stimuli can trigger counterregulatory expression of checkpoint molecules such as PD-L1. We have shown that a widely used clinical-grade DC maturation cocktail composed of TNF-α, IL-1β, IL-6, and PGE2 induces high levels of surface PD-L1, which rises even further after cryopreservation and thawing, an effect presumably due to the prostaglandin (87, 88).

The impact of the route of DC injection has also been insufficiently addressed so far. Many trials have used the i.d. or s.c. route of injection, as it is very safe and feasible. However, a disadvantage of this route of administration is that the majority of DCs remain stuck at the injection site and will fail to migrate to the T cell rich areas within draining lymph nodes (89). Direct intranodal administration of DCs has been tested in melanoma, however it is technically challenging, while being not superior in terms of evoked immune responses (90). A much more predictable biodistribution can be achieved by i.v. injection, where the totality of the dose encounters the pulmonary vascular bed first, with subsequent distribution to the spleen and liver, as shown in a unique imaging study on human subjects (91). Preclinical experiments have shown that the “lung vascular filter” acts as a site where robust T cell-mediated immune responses can be efficiently evoked (92). The fact that the human lung represents a reservoir of around 10 billion resident T cells (93) raises the likelihood of productive interactions with antigen-carrying DCs injected intravenously. In addition, the route of DC injection can have an impact on the trafficking pattern of primed T cells, as shown in preclinical studies (94). Whereas an s.c. injection will program elicited T cells to home towards the skin, i.v. injection of DCs induces splenic CD8+ T cells capable of homing towards hematogenic metastases, which is especially relevant for lung cancer. Interestingly, in a therapeutic DC vaccination phase 1 trial in advanced melanoma comparing several ratios of i.v. versus i.d. injection, the data suggest that the i.v. rather than the i.d. injection route could be pivotal for the exceptional objective responses observed (95). Importantly, despite the potential of activated DCs to secrete large amounts of inflammatory cytokines and chemokines, none of the trials using i.v. injection have observed life-threatening toxicity events. A different strategy is the intratumoral injection of DCs, the idea being that relevant tumor antigens are present in abundance, and that T cells can then be primed *in situ*. One study using *CCL21* gene-modified DCs delivered into lung tumors documented systemic antigen-specific CD8+ T cell responses in a fraction of the patients (52). This is surprising considering the strongly immunosuppressive microenvironment in lung tumors, shown to corrupt the function of DCs and T cells alike (96). Also, intratumoral injection is technically challenging and not feasible in every patient.

Finally, the optimal DC dose and injection schedule has not been determined yet. Given the complex and indirect mechanism of action of DC therapy and the imperfect nature of immune responses as surrogate for clinical responses, accurate modelling of a dose-effect relationship has not been achieved yet. What is clear, however, from all the early-phase trials, is that no dose-limiting AEs have been observed to date. Often the maximum dose delivered is practically limited by the production capacity of autologous DC vaccines. Still, one study suggested a better survival in NSCLC patients receiving the highest dose of DCs (54). Two other studies demonstrated a better OS in patients receiving respectively five and six or more vaccinations (48, 53). A bias may be present in these retrospective studies as the group of patients that received fewer vaccinations generally had a worse performance status.

## The Way Forward

### Optimizing the Choice of Target Antigens

The choice of target antigens for loading onto DCs is crucial to maximize the likelihood of eliciting a strong and tumor-directed immune response. Ideally, the antigen should meet different criteria: tumor-specific (expressed by cancer cells only), highly immunogenic, and necessary for cancer survival (97).

To date, most of the DC vaccination trials in lung cancer have targeted TAAs, which are self-antigens that are abnormally expressed by cancer cells, but may be present in normal cells as well. Since TAAs are shared with normal tissues, they can display limited immunogenicity due to central and peripheral tolerance, hence affecting the clinical efficacy of the vaccine. This can be partly circumvented by targeting more than one cancer antigen which will induce a broader immune response (98), as was the case in several of the abovementioned trials. However, the detectable immune responses that were evoked by the DC vaccine in these trials were often not powerful enough to translate into clinical effectiveness.

Development of personalized cancer vaccines based on neoantigens has become a new approach in cancer immunotherapy (99, 100). Neoantigens are tumor specific antigens that arise as a consequence of non-synonymous somatic mutations in the tumor cell genome (100). As their expression is tumor-restricted, in contrast to TAAs, these antigens are not subject to central tolerance and are potentially recognized by high avidity T cells. Hence, these antigens are ideal targets for DC vaccines. Neoantigens can be identified and selected using whole exome sequencing of tumor and blood cell DNA and bioinformatics algorithms. In a murine lung carcinoma model, neoantigen-pulsed DC vaccines were superior to neoantigen-adjuvant vaccines in activating immune responses and inhibiting tumor growth (101). A first demonstration of this approach in human subjects was provided in a phase 1 trial in metastatic melanoma, showing a remarkable induction of *de novo* T cell responses after vaccination with personalized tumor neoantigen-loaded DCs. Several phase 1 clinical trials are currently exploring neoantigen-targeted DC vaccines in lung cancer (**Table 3**), including a study from our group in surgically resected NSCLC (MIDRIX^NEO^-LUNG/NCT04078269), as well as in lung cancer patients who failed on standard anticancer therapies (NCT03871205). A major drawback for neoantigen-targeted vaccination is the lengthy and complex process leading up to the identification of potential neo-epitopes, which precludes implementation in patients with advanced or progressing disease. Also, the lack of standardization of *in silico* neo-epitope identification pipelines, with different algorithms producing diverging target lists, is a concern. One workaround consists of harvesting and profiling the “real” HLA-bound mutanome-derived peptides from tumor cells using advanced mass-spectrometric methods (102). This however requires access to large tumor samples which is a challenge in some clinical settings.

**Table 3:** Current clinical trials exploring dendritic cell (DC)-based immunotherapy in lung cancer.

Clinical trial I.D.	Study title	Interventions	Phase
NCT04078269	MIDRIXNEO-LUNG Dendritic Cell Vaccine in Patients With Non-small Cell Lung Cancer	Biological: Dendritic cell immunotherapyBiological: Antigen-specific DTHBiological: Control DTH	Phase 1
NCT04082182	MIDRIX4-LUNG Dendritic Cell Vaccine in Patients With Metastatic Non-small Cell Lung Cancer	Biological: Dendritic cell immunotherapyBiological: Antigen-specific DTHBiological: Control DTH	Phase 1
NCT03406715	Combination Immunotherapy-Ipilimumab-Nivolumab-Dendritic Cell p53 Vac – Patients With Small Cell Lung Cancer (SCLC)	Drug: NivolumabDrug: IpilimumabBiological: Dendritic Cell based p53 Vaccine	Phase 2
NCT04199559	Evaluating Combination Therapy using Autologous Dendritic Cells Pulsed With Antigen Peptides and Nivolumab for Subjects With Advanced Non-small Cell Lung Cancer	Drug: Autologous dendritic cells pulsed with antigen	Phase 1
NCT03371485	AST-VAC2 Vaccine in Patients With Non-small Cell Lung Cancer	Biological: AST-VAC2	Phase 1
NCT03360630	Anti-PD-1 Alone or Combined With Autologous Cell Therapy in Advanced NSCLC	Biological: Anti-PD-1 plus DC-CIKBiological: Anti-PD-1 alone	Phase 1
NCT03970746	Safety, Immunogenicity and Preliminary Clinical Activity Study of PDC*lung01 Cancer Vaccine in NSCLC	Biological: PDC*lung01Drug: Keytruda Injectable ProductDrug: Alimta Injectable Product	Phase 1/2
NCT04147078	Personalized DC Vaccine for Postoperative Cancer	Biological: DC vaccine subcutaneous administration	Phase 1
NCT03546361	Intratumoral Administration of CCL21-gene Modified Dendritic Cell With Intravenous Pembrolizumab for Advanced NSCLC	Genetic: Ad-CCL21-DC 1 × 10^7^ Genetic: Ad-CCL21-DC 3 × 10^7^ Genetic: Ad-CCL21-DC 0.05 × 10^7^Drug: Pembrolizumab Genetic: Ad-CCL21-DC ExD	Phase 1
NCT03735290	A study to Evaluate the Safety and Effectiveness of ILIxadencel Administered Into Tumors in Combination With Checkpoint Inhibitor (CPI) in Patients With Advanced Cancer	Biological: ilixadencelDrug: Pembrolizumab	Phase 1/2
NCT03047525	Study of DC-CTL Combined With CIK for Advanced Solid Tumor	Biological: Cytokine-induced Killer Cells	Phase 1/2
NCT02470468	Evaluation of Safety and Efficacy of DCVAC/LuCa (immunotherapy of Lung Cancer) in Patients With Metastatic Lung Cancer	Biological: DCVAC add on to SOCBiological: DCVAC and immune enhancers add on to SOCOther: Standard of Care Chemotherapy	Phase 1/2
NCT03871205	Neoantigen-primed DC Vaccines Therapy for Refractory Lung Cancer	Biological: Neoantigen loaded DC vaccine	Phase 1
NCT04147078	Personalized DC Vaccine for Postoperative Cancer	Biological: DC vaccine subcutaneous administration	Phase 1

Database search (ClinicalTrials.gov) was restricted to clinical trials that are active or will be activated in the near future.

### Selecting the Right Patients

Autologous cell therapies such as DC-based immunotherapies are labor-intensive and expensive to produce, and scaling-out to address a large patient population is difficult. Affordability of these therapies will be an important issue and challenge for both manufacturers and healthcare providers (103). Hence, a crucial question is which patients will derive most clinical benefit from these treatments. In early stage and locally advanced disease, treatable with curative intent (low tumor burden), the goal of DC-based immunotherapy is primarily to induce immunological memory to prevent later disease relapse (prophylactic vaccination). However, to show any therapeutic efficacy in this patient population, large and lengthy randomized trials are needed. In patients with metastatic disease on the other hand, DC-based immunotherapy actually aims to control the existing tumor (therapeutic vaccination). Considering the delayed antitumor effect and the systemic immunosuppression that is proportionate to the tumor load, patients with rapidly progressive or bulky tumors are unlikely to be appropriate candidates for DC vaccination, unless combinations with other systemic therapies are applied. In order to make DC therapy a viable option in clinical practice, biomarkers to enrich for responders/exclude non-responders upfront must imperatively be developed. Matching the targets loaded into the vaccine with the antigen expression pattern in the tumor is an obvious step. In addition, one can envision to exclude patients whose tumor biopsies harbor immune escape features such as loss-of-function or truncating mutations in Janus kinase (JAK) 1/2 or β2-microglobulin respectively, as vaccine-elicited T lymphocytes will fail to recognize and destroy the escape variants.

### Using the Right Combination Partner

Combination with other treatment modalities, such as chemotherapy, radiotherapy and especially immune checkpoint inhibition, may be the key to the success of DC-based immunotherapy and is currently the subject of several clinical trials (**Table 3**). In principle, all combinatorial strategies aiming to potentiate cancer vaccines in general are applicable to DC therapy in particular (see (18) for an extensive review).

The combination of DC therapy with chemotherapy may seem less suitable due to the immunosuppressive effects of the latter. However, it has become clear that cytotoxic drugs do also have several immune-potentiating effects, not only by inducing immunogenic cell death (104), but also by some ancillary effects on both cancer cells and immune cells present within the tumor3 microenvironment (TME) (105). Chemotherapy can for instance reduce systemic levels of MDSCs and Tregs, which are important factors of lymphocyte suppression in metastatic cancer patients. It was previously shown that vaccination in between platinum-containing chemotherapy cycles can indeed boost antigen-specific T cell responses (106), which is attributed to the MDSC-depleting effect of myelotoxic platinum salts. In addition, chemotherapeutics can also promote antitumor immune responses by upregulating the expression of tumor antigens and MHC class I molecules on the tumor, thereby increasing the capacity for antigen presentation (105). As such, chemotherapy could improve the efficacy of DC-based immunotherapy by rendering tumor cells more susceptible for immune-mediated killing elicited by the DC vaccine (107). Finally, different classes of chemotherapeutics can directly affect DC biology, resulting in upregulated costimulatory molecule expression and increased antigen presentation. For some chemotherapeutics such as taxanes, the effects are mediated by TLR triggering (108). To date, combinatorial approaches of chemotherapy and DC-based immunotherapy (mostly DC/CIK cell therapy) have been investigated only sporadically in lung cancer. A relevant and very recent study in NSCLC is SLU01, a phase 1/2 randomized, open-label, multicenter trial evaluating the clinical efficacy and safety of DCVAC/LuCa added to standard first-line chemotherapy (paclitaxel and carboplatin) and immune-enhancers (IFN-α and hydroxychloroquine) in patients with stage IV NSCLC (NCT02470468). Preliminary results, presented in abstract form (109), demonstrated a better OS in patients receiving the combination therapy versus chemotherapy alone (15.5 vs. 11.8 months, HR 0.55, 95% CI 0.33–0.93; p = 0.0232).

An emerging research topic is the complex interplay between radiotherapy (RT) and the immune system, since it was appreciated that RT can mediate tumor regression outside the radiation field. This phenomenon, called the “abscopal effect”, is shown to be the result of an immune-mediated mechanism (110, 111). The advent of immunotherapies, especially immune checkpoint inhibitors, has created special interest in strategies that combine RT with immunotherapeutic agents. RT can enhance systemic antitumor immune responses by several immunomodulatory mechanisms (112), which were already briefly mentioned earlier in this review. In this way, RT could act synergistically with DC-vaccination and thus improve clinical outcomes. Preclinical tumor models have indeed shown potent local and systemic antitumor responses when direct intratumoral administration of DCs was combined with RT (113, 114). The first modest signs of clinical efficacy in humans were demonstrated in small clinical trials involving patients with digestive tumors and high-risk soft tissue sarcomas (115–117), whereas evidence in lung cancer is limited only to some studies with DC/CIK cell therapy.

Given the spectacular emergence of immune checkpoint blockers (ICBs) in the lung cancer therapeutic landscape, questions inevitably arise as to the role of DC vaccination in this setting. Although ICBs, more specifically PD-1/PD-L1 inhibitors, can sometimes trigger dramatic durable responses, the majority of lung cancer patients still experiences disease progression within a year of treatment. This is not surprising given the fact that anti-PD-1 antibodies, the most commonly used ICBs in lung cancer, only “fix” one specific step in the cancer immunity cycle, which is alleviating T cell paralysis in the TME. Upstream of this, a whole sequence of events leading up to the induction of tumor-homing cytotoxic T cells is operated by DCs, which are known to be dysfunctional within cancer-bearing hosts. Hence, adoptive transfer of fully functional *ex vivo* generated DCs can be seen as an ideal complement to checkpoint inhibition, as a fitting illustration of “pushing the gas pedal” while also “releasing the brakes” (118). ICB failure is often a manifestation of an “immune cold” tumor, i.e., a phenotype characterized by a lack of T cell priming against tumor antigens and consequently an absence of tumor-infiltrating T cells. DC therapy can turn a “cold” into a “hot” tumor through its capacity to prime and generate a *de novo* tumor antigen-specific T cell population. In addition, expanding insights into the mechanisms of action of ICBs could help to design better DC-based therapeutic approaches. The emerging knowledge that exhausted T cells consist of a multi-stage and dynamic group of lymphocytes is extremely relevant in this context. Differences in abundance and distribution of these T cell subsets could underlie differential responsiveness to ICBs, as only “progenitor exhausted” T cells can be expanded by this therapy (119). It raises the question whether DC vaccination may replenish the immune system with the type of progenitor T cells that is amenable to rescue by anti-PD-1 blockade. New insights in the mechanism of action of anti-PD-L1 ICBs are also emerging, mostly diverting the traditional focus from T cell/cancer cell interactions in the TME. As recently reported, anti-PD-L1 ICBs may achieve much of its effect by blocking PD-L1/PD-1 interactions in lymph node-resident DC-T cell clusters, rather than at the level of the tumor (120). Also, adoptively transferred activated DCs express variable levels of surface PD-L1, such that the anti-PD-L1 combination partner must be judiciously chosen. On one hand the combination can indeed result in boosting of T cell responses. However PD-L1-blockade may also be detrimental to DC-mediated T cell priming as PD-L1 protects DCs from cytotoxic T cells during antigen-specific cognate interactions (121). At worse, an anti-PD-L1 ICB with a specific IgG subtype could in theory trigger elimination of the injected DCs through antibody-dependent cytotoxicity. Finally, although CTLA-4 blockade as such is not part of the standard-of-care in lung cancer, its capacity to boost T cell priming could make it an ideal partner in a DC-based combinatorial approach. Clinical evidence for this type of combination was already provided in a phase 1 trial in advanced melanoma patients, where a DC vaccine combined with ipilimumab resulted in remarkably high ORRs (122).

Clinical data supporting the combination of DC therapy and ICBs is not yet available in lung cancer, however several early-phase trials are already addressing this issue (NCT03406715, NCT03360630, NCT03970746, NCT03546361, and NCT03970746). Of these, PDC-LUNG-101 (NCT03970746) seems promising, evaluating the safety, clinical efficacy and immunogenicity of PDC*lung01, a peptide-pulsed allogeneic plasmacytoid DC line in combination with pembrolizumab in patients with metastatic NSCLC. Still many questions remain unanswered such as which class of immune checkpoint inhibition is most suited (anti-PD-1/PD-L1/CTLA4)?. Choosing the correct sequencing could also be critical as preclinical data suggest that PD-1 inhibition can induce a population of T cells that are refractory to subsequent stimulation by a vaccine (123). Additional factors may come into play as our understanding of ICB mechanism of action grows. Of note, accumulating data around the role of the gut microbiome in shaping responses to ICBs (124) may sooner or later impact the way we design cancer vaccination combinatorial studies, including DC immunotherapy.

## Conclusion

DC-based immunotherapy is safe and well-tolerated and can elicit antitumor immune responses in many patients with lung cancer, with occasional yet remarkable objective responses despite the predominant immunosuppressive climate in the metastatic setting. Combining DC-based immunotherapy with other anticancer therapies, such as chemotherapy, radiotherapy and/or checkpoint inhibition, can potentially improve their effectiveness. Alternatively, a choice of antigens based on neoepitopes with proven expression by the tumor cells may not merely induce immune responses but could result in clinical responses. Clinical trials to prove these hypotheses are underway and the results are eagerly awaited. Additional challenges for the future of DC therapy are determining the adequate dose, frequency, and duration of treatment, improving the choice of target antigens, and finding biomarkers to select potential responders upfront. Finally, identifying the most synergistic combinatorial regimen can hold the real key to long term disease control and survival in this lethal disease.

## Author Contributions

DS and KV wrote the manuscript. JI, SVL and BV read and corrected where needed. DS and KV took part in the discussion leading up to the manuscript. All authors contributed to the article and approved the submitted version.

## Funding

KV is supported by an FWO Senior Clinical Investigator Grant. JI received a University BOF (Bijzonder Onderzoeksfonds) grant.

## Conflict of Interest

The authors declare that the research was conducted in the absence of any commercial or financial relationships that could be construed as a potential conflict of interest.

## Abbreviations

Ad, adenovirus; AE, adverse event; AKT, activated killer T cell; APC, antigen presenting cell; ATRA, all-trans retinoic acid; CEA, carcinoembryonic antigen; CIK, cytokine-induced killer cell; CR, complete response; CRC, colorectal cancer; CRT, chemoradiotherapy; CTA, cancer-testis antigen; CTL, cytotoxic T lymphocyte; CTLA-4, cytotoxic T lymphocyte-associated antigen 4; DC, dendritic cell; DCTCMF, dendritic cell/T cell-derived maturation factor; DTH, delayed-type hypersensitivity; ELISPOT, enzyme-linked immune absorbent spot; G-CSF, granulocyte colony-stimulating factor; GM-CSF, granulocyte/macrophage colony-stimulating factor; HR, hazard ratio; HER2, human epidermal growth factor receptor 2; HLA, human leucocyte antigen; ICB, immune checkpoint blocker/blockade; IFN, interferon; IL, interleukin; i.d., intradermal; i.v., intravenous; JAK, Janus kinase; KHL, keyhole limpet hemocyanin; MAGE, melanoma Antigen; MDSC, myeloid-derived suppressor cell; MHC, major histocompatibility complex; MST, median survival time; MUC1, mucin 1; NSCLC, non-small cell lung cancer; ORR, objective response rate; OS, overall survival; PBMC, peripheral blood monocyte; PD, progressive disease; PD-1, programmed cell death protein 1; PD-L1, programmed death-ligand 1; PFS, progression-free survival; PGE2, prostaglandin E2; PR, partial response; QOL, quality of life; RCT, randomized controlled trial; RT, radiotherapy; s.c., subcutaneous; SCLC, small cell lung cancer; SD, stable disease; SOCS1, suppressor of cytokine signaling 1; TAA, tumor-associated antigens; TLR, toll-like receptor; TME, tumor microenvironment; TNF, tumor necrosis factor; TNM, tumor node metastasis; Treg, t-regulatory; TRT, thoracic radiotherapy; WT1, wilms tumor protein 1.

## References

[1] Bray F Ferlay J Soerjomataram I Siegel RL Torre LA Jemal A. Global cancer statistics 2018: GLOBOCAN estimates of incidence and mortality worldwide for 36 cancers in 185 countries. CA: A Cancer J Clin (2018) 68:394–424. 10.3322/caac.21492 30207593

[2] Siegel RL Miller KD Jemal A. Cancer statistics, 2018. CA: A Cancer J Clin (2018) 68:7–30. 10.3322/caac.21442 29313949

[3] Reck M Rodriguez-Abreu D Robinson AG Hui R Csoszi T Fulop A. Pembrolizumab versus Chemotherapy for PD-L1-Positive Non-Small-Cell Lung Cancer. N Engl J Med (2016) 375:1823–33. 10.1056/NEJMoa1606774 27718847

[4] Gandhi L Rodríguez-Abreu D Gadgeel S Esteban E Felip E De Angelis F. Pembrolizumab plus Chemotherapy in Metastatic Non–Small-Cell Lung Cancer. N Engl J Med (2018) 378:2078–92. 10.1056/NEJMoa1801005 29658856

[5] Horn L Mansfield AS Szczęsna A Havel L Krzakowski M Hochmair MJ. First-Line Atezolizumab plus Chemotherapy in Extensive-Stage Small-Cell Lung Cancer. N Engl J Med (2018) 379:2220–9. 10.1056/NEJMoa1809064 30280641

[6] Paz-Ares L Luft A Vicente D Tafreshi A Gümüş M Mazières J. Pembrolizumab plus Chemotherapy for Squamous Non–Small-Cell Lung Cancer. N Engl J Med (2018) 379:2040–51. 10.1056/NEJMoa1810865 30280635

[7] Paz-Ares L Dvorkin M Chen Y Reinmuth N Hotta K Trukhin D. Durvalumab plus platinum-etoposide versus platinum-etoposide in first-line treatment of extensive-stage small-cell lung cancer (CASPIAN): a randomised, controlled, open-label, phase 3 trial. Lancet (2019) 394:1929–39. 10.1016/S0140-6736(19)32222-6 31590988

[8] Borghaei H Paz-Ares L Horn L Spigel DR Steins M Ready NE. Nivolumab versus Docetaxel in Advanced Nonsquamous Non-Small-Cell Lung Cancer. N Engl J Med (2015) 373:1627–39. 10.1056/NEJMoa1507643 PMC5705936 26412456

[9] Brahmer J Reckamp KL Baas P Crino L Eberhardt WE Poddubskaya E. Nivolumab versus Docetaxel in Advanced Squamous-Cell Non-Small-Cell Lung Cancer. N Engl J Med (2015) 373:123–35. 10.1056/NEJMoa1504627 PMC4681400 26028407

[10] Herbst RS Baas P Kim DW Felip E Perez-Gracia JL Han JY. Pembrolizumab versus docetaxel for previously treated, PD-L1-positive, advanced non-small-cell lung cancer (KEYNOTE-010): a randomised controlled trial. Lancet (2016) 387:1540–50. 10.1016/S0140-6736(15)01281-7 26712084

[11] Rittmeyer A Barlesi F Waterkamp D Park K Ciardiello F Von Pawel J. Atezolizumab versus docetaxel in patients with previously treated non-small-cell lung cancer (OAK): a phase 3, open-label, multicentre randomised controlled trial. Lancet (2017) 389:255–65. 10.1016/S0140-6736(16)32517-X PMC6886121 27979383

[12] Muenst S Laubli H Soysal SD Zippelius A Tzankov A Hoeller S. The immune system and cancer evasion strategies: therapeutic concepts. J Intern Med (2016) 279:541–62. 10.1111/joim.12470 26748421

[13] O’Donnell JS Teng MWL Smyth MJ. Cancer immunoediting and resistance to T cell-based immunotherapy. Nat Rev Clin Oncol (2019) 16:151–67. 10.1038/s41571-018-0142-8 30523282

[14] van der Burg SH Arens R Ossendorp F van Hall T Melief CJ. Vaccines for established cancer: overcoming the challenges posed by immune evasion. Nat Rev Cancer (2016) 16:219–33. 10.1038/nrc.2016.16 26965076

[15] Villanueva N Bazhenova L. New strategies in immunotherapy for lung cancer: beyond PD-1/PD-L1. Ther Adv Respir Dis (2018) 12:1–29. 10.1177/1753466618794133 PMC6144513 30215300

[16] Albright C Garst J. Vaccine therapy in non—small-cell lung cancer. Curr Oncol Rep (2007) 9:241–6. 10.1007/s11912-007-0029-9 17588347

[17] Kalinski P Urban J Narang R Berk E Wieckowski E Muthuswamy R. Dendritic cell-based therapeutic cancer vaccines: what we have and what we need. Future Oncol (2009) 5:379–90. 10.2217/fon.09.6 PMC2713774 19374544

[18] Vermaelen K. Vaccine Strategies to Improve Anti-cancer Cellular Immune Responses. Front Immunol (2019) 10:8. 10.3389/fimmu.2019.00008 30723469 PMC6349827

[19] Brunsvig PF Kyte JA Kersten C Sundstrom S Moller M Nyakas M. Telomerase Peptide Vaccination in NSCLC: A Phase II Trial in Stage III Patients Vaccinated after Chemoradiotherapy and an 8-Year Update on a Phase I/II Trial. Clin Cancer Res (2011) 17:6847–57. 10.1158/1078-0432.CCR-11-1385 21918169

[20] Butts C Socinski MA Mitchell PL Thatcher N Havel L Krzakowski M. Tecemotide (L-BLP25) versus placebo after chemoradiotherapy for stage III non-small-cell lung cancer (START): a randomised, double-blind, phase 3 trial. Lancet Oncol (2014) 15:59–68. 10.1016/S1470-2045(13)70510-2 24331154

[21] Nemunaitis J Jahan T Ross H Sterman D Richards D Fox B. Phase 1/2 trial of autologous tumor mixed with an allogeneic GVAX^®^ vaccine in advanced-stage non-small-cell lung cancer. Cancer Gene Ther (2006) 13:555–62. 10.1038/sj.cgt.7700922 16410826

[22] Vansteenkiste JF Cho BC Vanakesa T De Pas T Zielinski M Kim MS. Efficacy of the MAGE-A3 cancer immunotherapeutic as adjuvant therapy in patients with resected MAGE-A3-positive non-small-cell lung cancer (MAGRIT): a randomised, double-blind, placebo-controlled, phase 3 trial. Lancet Oncol (2016) 17:822–35. 10.1016/S1470-2045(16)00099-1 27132212

[23] Thomas A Giaccone G. Why has active immunotherapy not worked in lung cancer? Ann Oncol (2015) 26:2213–20. 10.1093/annonc/mdv323 PMC4621028 26232492

[24] Steinman RM Cohn ZA. Identification of a novel cell type in peripheral lymphoid organs of mice. J Exp Med (1973) 137:1142–62. 10.1084/jem.137.5.1142 PMC2139237 4573839

[25] Melief CJM. Cancer Immunotherapy by Dendritic Cells. Immunity (2008) 29:372–83. 10.1016/j.immuni.2008.08.004 18799145

[26] Steinman RM Banchereau J. Taking dendritic cells into medicine. Nature (2007) 449:419–26. 10.1038/nature06175 17898760

[27] Palucka K Banchereau J. Cancer immunotherapy *via* dendritic cells. Nat Rev Cancer (2012) 12:265–77. 10.1038/nrc3258 PMC3433802 22437871

[28] Schuler G Schuler-Thurner B Steinman RM. The use of dendritic cells in cancer immunotherapy. Curr Opin Immunol (2003) 15:138–47. 10.1016/s0952-7915(03)00015-3 12633662

[29] Cranmer LD Trevor KT Hersh EM. Clinical applications of dendritic cell vaccination in the treatment of cancer. Cancer Immunol Immunother (2004) 53:275–306. 10.1007/s00262-003-0432-5 14648069 PMC11032969

[30] Wimmers F Schreibelt G Skãld AE Figdor CG De Vries IJM. Paradigm Shift in Dendritic Cell-Based Immunotherapy: From *in vitro* Generated Monocyte-Derived DCs to Naturally Circulating DC Subsets. Front Immunol (2014) 5:165. 10.3389/fimmu.2014.00165 24782868 PMC3990057

[31] Huber A Dammeijer F Aerts JGJV Vroman H. Current State of Dendritic Cell-Based Immunotherapy: Opportunities for *in vitro* Antigen Loading of Different DC Subsets? Front Immunol (2018) 9:2804. 10.3389/fimmu.2018.02804 30559743 PMC6287551

[32] Anguille S Smits EL Lion E van Tendeloo VF Berneman ZN. Clinical use of dendritic cells for cancer therapy. Lancet Oncol (2014) 15:e257–67. 10.1016/S1470-2045(13)70585-0 24872109

[33] Carreno BM Magrini V Becker-Hapak M Kaabinejadian S Hundal J Petti AA. A dendritic cell vaccine increases the breadth and diversity of melanoma neoantigen-specific T cells. Science (2015) 348:803–8. 10.1126/science.aaa3828 PMC4549796 25837513

[34] Draube A Klein-González N Mattheus S Brillant C Hellmich M Engert A. Dendritic Cell Based Tumor Vaccination in Prostate and Renal Cell Cancer: A Systematic Review and Meta-Analysis. PLoS One (2011) 6:e18801. 10.1371/journal.pone.0018801 21533099 PMC3080391

[35] Fong L Hou Y Rivas A Benike C Yuen A Fisher GA. Altered peptide ligand vaccination with Flt3 ligand expanded dendritic cells for tumor immunotherapy. Proc Natl Acad Sci (2001) 98:8809–14. 10.1073/pnas.141226398 PMC37517 11427731

[36] Itoh T Ueda Y Kawashima I Nukaya I Fujiwara H Fuji N. Immunotherapy of solid cancer using dendritic cells pulsed with the HLA-A24-restricted peptide of carcinoembryonic antigen. Cancer Immunol Immunother (2002) 51:99–106. 10.1007/s00262-001-0257-z 11904734 PMC11032765

[37] Nair SK Morse M Boczkowski D Ian Cumming R Vasovic L Gilboa E. Induction of Tumor-Specific Cytotoxic T Lymphocytes in Cancer Patients by Autologous Tumor RNA-Transfected Dendritic Cells. Ann Surg (2002) 235:540–9. 10.1097/00000658-200204000-00013 PMC1422470 11923611

[38] Kontani K Taguchi O Ozaki Y Hanaoka J Sawai S Inoue S. Dendritic cell vaccine immunotherapy of cancer targeting MUC1 mucin. Int J Mol Med (2003) 12:493–502. 10.3892/ijmm.12.4.493 12964025

[39] Hirschowitz EA. Autologous Dendritic Cell Vaccines for Non-Small-Cell Lung Cancer. J Clin Oncol (2004) 22:2808–15. 10.1200/JCO.2004.01.074 15254048

[40] Yannelli JR Sturgill J Foody T Hirschowitz E. The large scale generation of dendritic cells for the immunization of patients with non-small cell lung cancer (NSCLC). Lung Cancer (2005) 47:337–50. 10.1016/j.lungcan.2004.08.008 15713517

[41] Ueda Y Itoh T Nukaya I Kawashima I Okugawa K Yano Y. Dendritic cell-based immunotherapy of cancer with carcinoembryonic antigen-derived, HLA-A24-restricted CTL epitope: Clinical outcomes of 18 patients with metastatic gastrointestinal or lung adenocarcinomas. Int J Oncol (2004) 24:909–17. 10.3892/ijo.24.4.909 15010829

[42] Chang G-C Lan H-C Juang S-H Wu Y-C Lee H-C Hung Y-M. A pilot clinical trial of vaccination with dendritic cells pulsed with autologous tumor cells derived from malignant pleural effusion in patients with late-stage lung carcinoma. Cancer (2005) 103:763–71. 10.1002/cncr.20843 15637694

[43] Morse MA. Phase I Study of Immunization with Dendritic Cells Modified with Fowlpox Encoding Carcinoembryonic Antigen and Costimulatory Molecules. Clin Cancer Res (2005) 11:3017–24. 10.1158/1078-0432.CCR-04-2172 15837756

[44] Hirschowitz EA Foody T Hidalgo GE Yannelli JR. Immunization of NSCLC patients with antigen-pulsed immature autologous dendritic cells. Lung Cancer (2007) 57:365–72. 10.1016/j.lungcan.2007.04.002 PMC2063443 17509725

[45] Mayordomo JI Andres R Isla MD Murillo L Cajal R Yubero A. Results of a pilot trial of immunotherapy with dendritic cells pulsed with autologous tumor lysates in patients with advanced cancer. Tumori (2007) 93:26–30. 10.1177/030089160709300106 17455868

[46] Um S-J Choi YJ Shin H-J Son CH Park Y-S Roh MS. Phase I study of autologous dendritic cell tumor vaccine in patients with non-small cell lung cancer. Lung Cancer (2010) 70:188–94. 10.1016/j.lungcan.2010.02.006 20223553

[47] Perroud MW Honma HN Barbeiro AS Gilli SC Almeida MT Vassallo J. Mature autologous dendritic cell vaccines in advanced non-small cell lung cancer: a phase I pilot study. Journal of Experimental & Clinical Cancer Research (2011) 30:65. 10.1186/1756-9966-30-65 PMC3135553 21682877

[48] Takahashi H Okamoto M Shimodaira S Tsujitani S-I Nagaya M Ishidao T. Impact of dendritic cell vaccines pulsed with Wilms’ tumour-1 peptide antigen on the survival of patients with advanced non-small cell lung cancers. Eur J Cancer (2013) 49:852–9. 10.1016/j.ejca.2012.11.005 23245331

[49] Hu RH Shi SB Qi JL Tian J Tang XY Liu GF. Pemetrexed plus dendritic cells as second-line treatment for patients with stage IIIB/IV non-small cell lung cancer who had treatment with TKI. Med Oncol (2014) 31:63. 10.1007/s12032-014-0063-z 24958515

[50] Takahashi H Shimodaira S Ogasawara M Ota S Kobayashi M Abe H. Lung adenocarcinoma may be a more susceptive subtype to a dendritic cell-based cancer vaccine than other subtypes of non-small cell lung cancers: a multicenter retrospective analysis. Cancer Immunol Immunother (2016) 65:1099–111. 10.1007/s00262-016-1872-z PMC11029687 27448677

[51] Li D He S. MAGE3 and Survivin activated dendritic cell immunotherapy for the treatment of non-small cell lung cancer. Oncol Lett (2018) 15:8777–83. 10.3892/ol.2018.8362 PMC5950540 29805617

[52] Lee JM Lee M-H Garon E Goldman JW Salehi-Rad R Baratelli FE. Phase I Trial of Intratumoral Injection ofCCL21Gene–Modified Dendritic Cells in Lung Cancer Elicits Tumor-Specific Immune Responses and CD8+T-cell Infiltration. Clin Cancer Res (2017) 23:4556–68. 10.1158/1078-0432.CCR-16-2821 PMC5599263 28468947

[53] Teramoto K Ozaki Y Hanaoka J Sawai S Tezuka N Fujino S. Predictive biomarkers and effectiveness of MUC1-targeted dendritic-cell-based vaccine in patients with refractory non-small cell lung cancer. Ther Adv Med Oncol (2017) 9:147–57. 10.1177/1758834016678375 PMC5349424 28344660

[54] Ge C Li R Song H Geng T Yang J Tan Q. Phase I clinical trial of a novel autologous modified-DC vaccine in patients with resected NSCLC. BMC Cancer (2017) 17:884. 10.1186/s12885-017-3859-3 29268708 PMC5740508

[55] Li H Wang C Yu J Cao S Wei F Zhang W. Dendritic cell-activated cytokine-induced killer cells enhance the anti-tumor effect of chemotherapy on non-small cell lung cancer in patients after surgery. Cytotherapy (2009) 11:1076–83. 10.3109/14653240903121252 19929470

[56] Zhong R Teng J Han B Zhong H. Dendritic cells combining with cytokine-induced killer cells synergize chemotherapy in patients with late-stage non-small cell lung cancer. Cancer Immunol Immunother (2011) 60:1497–502. 10.1007/s00262-011-1060-0 PMC11029021 21681372

[57] Shi SB Ma TH Li CH Tang XY. Effect of maintenance therapy with dendritic cells: cytokine-induced killer cells in patients with advanced non-small cell lung cancer. Tumori (2012) 98:314–9. 10.1700/1125.12398 22825506

[58] Yang L Ren B Li H Yu J Cao S Hao X. Enhanced antitumor effects of DC-activated CIKs to chemotherapy treatment in a single cohort of advanced non-small-cell lung cancer patients. Cancer Immunol Immunother (2013) 62:65–73. 10.1007/s00262-012-1311-8 22744010 PMC11028994

[59] Gao X Mi Y Guo N Xu H Xu L Gou X. Cytokine-Induced Killer Cells As Pharmacological Tools for Cancer Immunotherapy. Front Immunol (2017) 8:774. 10.3389/fimmu.2017.00774 28729866 PMC5498561

[60] Zhao P Bu X Wei X Sun W Xie X Li C. Dendritic cell immunotherapy combined with cytokine-induced killer cells promotes skewing toward Th2 cytokine profile in patients with metastatic non-small cell lung cancer. Int Immunopharmacol (2015) 25:450–6. 10.1016/j.intimp.2015.02.010 25698555

[61] Zhu XP Xu YH Zhou J Pan XF. A clinical study evaluating dendritic and cytokine-induced killer cells combined with concurrent radiochemotherapy for stage IIIB non-small cell lung cancer. Genet Mol Res (2015) 14:10228–35. 10.4238/2015.August.28.6 26345959

[62] Zhang L Yang X Sun Z Li J Zhu H Li J. Dendritic cell vaccine and cytokine-induced killer cell therapy for the treatment of advanced non-small cell lung cancer. Oncol Lett (2016) 11:2605–10. 10.3892/ol.2016.4273 PMC4812113 27073525

[63] Zhang L Xu Y Shen J He F Zhang D Chen Z. Feasibility study of DCs/CIKs combined with thoracic radiotherapy for patients with locally advanced or metastatic non-small-cell lung cancer. Radiat Oncol (2016) 11:60. 10.1186/s13014-016-0635-5 27097970 PMC4839093

[64] Song H Liu S Zhao Z Sun W Wei X Ma X. Increased cycles of DC/CIK immunotherapy decreases frequency of Tregs in patients with resected NSCLC. Int Immunopharmacol (2017) 52:97–202. 10.1016/j.intimp.2017.09.014 28941416

[65] Chen C-L Pan Q-Z Weng D-S Xie C-M Zhao J-J Chen M-S. Safety and activity of PD-1 blockade-activated DC-CIK cells in patients with advanced solid tumors. OncoImmunology (2018) 7:e1417721. 10.1080/2162402X.2017.1417721 29632736 PMC5889206

[66] Kimura H Iizasa T Ishikawa A Shingyouji M Yoshino M Kimura M. Prospective phase II study of post-surgical adjuvant chemo-immunotherapy using autologous dendritic cells and activated killer cells from tissue culture of tumor-draining lymph nodes in primary lung cancer patients. Anticancer Res (2008) 28:1229–38. 18505060

[67] Kimura H Matsui Y Ishikawa A Nakajima T Iizasa T. Randomized controlled phase III trial of adjuvant chemoimmunotherapy with activated cytotoxic T cells and dendritic cells from regional lymph nodes of patients with lung cancer. Cancer Immunol Immunother (2018) 67:1231–8. 10.1007/s00262-018-2180-6 PMC6097784 29855695

[68] Kimura H Matsui Y Ishikawa A Nakajima T Yoshino M Sakairi Y. Randomized controlled phase III trial of adjuvant chemo-immunotherapy with activated killer T cells and dendritic cells in patients with resected primary lung cancer. Cancer Immunol Immunother (2015) 64:51–9. 10.1007/s00262-014-1613-0 PMC4282697 25262164

[69] Chiappori AA Soliman H Janssen WE Antonia SJ Gabrilovich DI. INGN-225: a dendritic cell-based p53 vaccine (Ad.p53-DC) in small cell lung cancer: observed association between immune response and enhanced chemotherapy effect. Expert Opin Biol Ther (2010) 10:983–91. 10.1517/14712598.2010.484801 PMC3146364 20420527

[70] Chiappori AA Williams CC Gray JE Tanvetyanon T Haura EB Creelan BC. Randomized-controlled phase II trial of salvage chemotherapy after immunization with a TP53-transfected dendritic cell-based vaccine (Ad.p53-DC) in patients with recurrent small cell lung cancer. Cancer Immunol Immunother (2019) 68:517–27. 10.1007/s00262-018-2287-9 PMC6426813 30591959

[71] Iclozan C Antonia S Chiappori A Chen D-T Gabrilovich D. Therapeutic regulation of myeloid-derived suppressor cells and immune response to cancer vaccine in patients with extensive stage small cell lung cancer. Cancer Immunol Immunother (2013) 62:909–18. 10.1007/s00262-013-1396-8 PMC3662237 23589106

[72] Shi SB Tang XY Tian J Chang CX Li P Qi JL. Efficacy of erlotinib plus dendritic cells and cytokine-induced killer cells in maintenance therapy of advanced non-small cell lung cancer. J Immunother (2014) 37:250–5. 10.1097/CJI.0000000000000015 24714359

[73] Cascio S Finn O. Intra- and Extra-Cellular Events Related to Altered Glycosylation of MUC1 Promote Chronic Inflammation, Tumor Progression, Invasion, and Metastasis. Biomolecules (2016) 6:39. 10.3390/biom6040039 PMC5197949 27754373

[74] Quoix E Lena H Losonczy G Forget F Chouaid C Papai Z. TG4010 immunotherapy and first-line chemotherapy for advanced non-small-cell lung cancer (TIME): results from the phase 2b part of a randomised, double-blind, placebo-controlled, phase 2b/3 trial. Lancet Oncol (2016) 17:212–23. 10.1016/S1470-2045(15)00483-0 26727163

[75] Thistlethwaite FC Gilham DE Guest RD Rothwell DG Pillai M Burt DJ. The clinical efficacy of first-generation carcinoembryonic antigen (CEACAM5)-specific CAR T cells is limited by poor persistence and transient pre-conditioning-dependent respiratory toxicity. Cancer Immunol Immunother (2017) 66:1425–36. 10.1007/s00262-017-2034-7 PMC5645435 28660319

[76] Hanna N Shepherd FA Fossella FV Pereira JR De Marinis F von Pawel J. Randomized phase III trial of pemetrexed versus docetaxel in patients with non-small-cell lung cancer previously treated with chemotherapy. J Clin Oncol (2004) 22:1589–97. 10.1200/JCO.2004.08.163 15117980

[77] Wang S Wang Z. Efficacy and safety of dendritic cells co-cultured with cytokine-induced killer cells immunotherapy for non-small-cell lung cancer. Int Immunopharmacol (2015) 28:22–8. 10.1016/j.intimp.2015.05.021 26013109

[78] Antonia SJ Villegas A Daniel D Vicente D Murakami S Hui R. Overall Survival with Durvalumab after Chemoradiotherapy in Stage III NSCLC. N Engl J Med (2018) 379:2342–50. 10.1056/NEJMoa1809697 30280658

[79] Kimura H Dobrenkov K Iida T Suzuki M Ando S Yamamoto N. Tumor-draining lymph nodes of primary lung cancer patients: a potent source of tumor-specific killer cells and dendritic cells. Anticancer Res (2005) 25:85–94. 15816523

[80] Antonia SJ López-Martin JA Bendell J Ott PA Taylor M Eder JP. Nivolumab alone and nivolumab plus ipilimumab in recurrent small-cell lung cancer (CheckMate 032): a multicentre, open-label, phase 1/2 trial. Lancet Oncol (2016) 17:883–95. 10.1016/S1470-2045(16)30098-5 27269741

[81] Chung HC Piha-Paul SA Lopez-Martin J Schellens JHM Kao S Miller WH Jr. Pembrolizumab After Two or More Lines of Previous Therapy in Patients With Recurrent or Metastatic SCLC: Results From the KEYNOTE-028 and KEYNOTE-158 Studies. J Thorac Oncol (2020) 15:618–27. 10.1016/j.jtho.2019.12.109 31870883

[82] Antonia SJ Mirza N Fricke I Chiappori A Thompson P Williams N. Combination of p53 cancer vaccine with chemotherapy in patients with extensive stage small cell lung cancer. Clin Cancer Res (2006) 12:878–87. 10.1158/1078-0432.CCR-05-2013 16467102

[83] Smit EF Fokkema E Biesma B Groen HJ Snoek W Postmus PE. A phase II study of paclitaxel in heavily pretreated patients with small-cell lung cancer. Br J Cancer (1998) 77:347–51. 10.1038/bjc.1998.54 PMC2151229 9461009

[84] Yamamoto N Tsurutani J Yoshimura N Asai G Moriyama A Nakagawa K. Phase II study of weekly paclitaxel for relapsed and refractory small cell lung cancer. Anticancer Res (2006) 26:777–81. 16739353

[85] Aarntzen EHJG Figdor CG Adema GJ Punt CJA De Vries IJM. Dendritic cell vaccination and immune monitoring. Cancer Immunol Immunother (2008) 57:1559–68. 10.1007/s00262-008-0553-y PMC2491428 18618110

[86] Lesterhuis WJ De Vries IJM Schuurhuis DH Boullart ACI Jacobs JFM De Boer AJ. Vaccination of colorectal cancer patients with CEA-loaded dendritic cells: antigen-specific T cell responses in DTH skin tests. Ann Oncol (2006) 17:974–80. 10.1093/annonc/mdl072 16600979

[87] Brabants E Heyns K De Smet S Devreker P Ingels J De Cabooter N. An accelerated, clinical-grade protocol to generate high yields of type 1-polarizing messenger RNA-loaded dendritic cells for cancer vaccination. Cytotherapy (2018) 20:1164–81. 10.1016/j.jcyt.2018.06.006 30122654

[88] Prima V Kaliberova LN Kaliberov S Curiel DT Kusmartsev S. COX2/mPGES1/PGE2pathway regulates PD-L1 expression in tumor-associated macrophages and myeloid-derived suppressor cells. Proc Natl Acad Sci (2017) 114:1117–22. 10.1073/pnas.1424355112 PMC5293015 28096371

[89] Verdijk P Aarntzen EH Lesterhuis WJ Boullart AC Kok E van Rossum MM. Limited amounts of dendritic cells migrate into the T-cell area of lymph nodes but have high immune activating potential in melanoma patients. Clin Cancer Res (2009) 15:2531–40. 10.1158/1078-0432.CCR-08-2729 19318472

[90] Bol KF Figdor CG Aarntzen EH Welzen ME Van Rossum MM Blokx WA. Intranodal vaccination with mRNA-optimized dendritic cells in metastatic melanoma patients. OncoImmunology (2015) 4:e1019197. 10.1080/2162402X.2015.1019197 26405571 PMC4570143

[91] Morse MA Coleman RE Akabani G Niehaus N Coleman D Lyerly HK. Migration of human dendritic cells after injection in patients with metastatic malignancies. Cancer Res (1999) 59:56–8. 9892184

[92] Willart MA Jan de Heer H Hammad H Soullie T Deswarte K Clausen BE. The lung vascular filter as a site of immune induction for T cell responses to large embolic antigen. J Exp Med (2009) 206:2823–35. 10.1084/jem.20082401 PMC2806611 19858325

[93] Purwar R Campbell J Murphy G Richards WG Clark RA Kupper TS. Resident memory T cells (T(RM)) are abundant in human lung: diversity, function, and antigen specificity. PLoS One (2011) 6:e16245. 10.1371/journal.pone.0016245 21298112 PMC3027667

[94] Mullins DW Sheasley SL Ream RM Bullock TN Fu YX Engelhard VH. Route of immunization with peptide-pulsed dendritic cells controls the distribution of memory and effector T cells in lymphoid tissues and determines the pattern of regional tumor control. J Exp Med (2003) 198:1023–34. 10.1084/jem.20021348 PMC2194213 14530375

[95] Wilgenhof S Van Nuffel AM Benteyn D Corthals J Aerts C Heirman C. A phase IB study on intravenous synthetic mRNA electroporated dendritic cell immunotherapy in pretreated advanced melanoma patients. Ann Oncol (2013) 24:2686–93. 10.1093/annonc/mdt245 23904461

[96] Pyfferoen L Brabants E Everaert C De Cabooter N Heyns K Deswarte K. The transcriptome of lung tumor-infiltrating dendritic cells reveals a tumor-supporting phenotype and a microRNA signature with negative impact on clinical outcome. Oncoimmunology (2017) 6:e1253655. 10.1080/2162402X.2016.1253655 28197369 PMC5283643

[97] Hollingsworth RE Jansen K. Turning the corner on therapeutic cancer vaccines. NPJ Vaccines (2019) 4:7. 10.1038/s41541-019-0103-y 30774998 PMC6368616

[98] Lybaert L Vermaelen K De Geest BG Nuhn L. Immunoengineering through cancer vaccines - A personalized and multi-step vaccine approach towards precise cancer immunity. J Control Rel (2018) 289:125–45. 10.1016/j.jconrel.2018.09.009 30223044

[99] Mastelic-Gavillet B Balint K Boudousquie C Gannon PO Kandalaft LE. Personalized Dendritic Cell Vaccines—Recent Breakthroughs and Encouraging Clinical Results. Front Immunol (2019) 10:766. 10.3389/fimmu.2019.00766 31031762 PMC6470191

[100] Peng M Mo Y Wang Y Wu P Zhang Y Xiong F. Neoantigen vaccine: an emerging tumor immunotherapy. Mol Cancer (2019) 18:128. 10.1186/s12943-019-1055-6 31443694 PMC6708248

[101] Zhang R Yuan F Shu Y Tian Y Zhou B Yi L. Personalized neoantigen-pulsed dendritic cell vaccines show superior immunogenicity to neoantigen-adjuvant vaccines in mouse tumor models. Cancer Immunol Immunother (2020) 69:135–45. 10.1007/s00262-019-02448-z PMC6949210 31807878

[102] Ebrahimi-Nik H Michaux J Corwin WL Keller GL Shcheglova T Pak H. Mass spectrometry driven exploration reveals nuances of neoepitope-driven tumor rejection. JCI Insight (2019) 5:e129152. 10.1172/jci.insight.129152 PMC6675551 31219806

[103] Lopes AG SA Frohlich B. Cost Analysis of Cell Therapy Manufacture: Autologous Cell Therapies, Part 1. BioProcess Int (2018) 16:S3–8.

[104] Ghiringhelli F Apetoh L Tesniere A Aymeric L Ma Y Ortiz C. Activation of the NLRP3 inflammasome in dendritic cells induces IL-1beta-dependent adaptive immunity against tumors. Nat Med (2009) 15:1170–8. 10.1038/nm.2028 19767732

[105] Chen G Emens LA. Chemoimmunotherapy: reengineering tumor immunity. Cancer Immunol Immunother (2013) 62:203–16. 10.1007/s00262-012-1388-0 PMC3608094 23389507

[106] Welters MJ van der Sluis TC van Meir H Loof NM van Ham VJ van Duikeren S. Vaccination during myeloid cell depletion by cancer chemotherapy fosters robust T cell responses. Sci Transl Med (2016) 8:334ra52. 10.1126/scitranslmed.aad8307 27075626

[107] van Gulijk M Dammeijer F Aerts J Vroman H. Combination Strategies to Optimize Efficacy of Dendritic Cell-Based Immunotherapy. Front Immunol (2018) 9:2759. 10.3389/fimmu.2018.02759 30568653 PMC6289976

[108] Truxova I Hensler M Skapa P Halaska MJ Laco J Ryska A. Rationale for the Combination of Dendritic Cell-Based Vaccination Approaches With Chemotherapy Agents. Int Rev Cell Mol Biol (2017) 330:115–56. 10.1016/bs.ircmb.2016.09.003 28215530

[109] Havel L Kolek V Pesek M Cernovská M Bartunkova J Spisek R. Dendritic-cell vaccine (DCVAC) with first-line chemotherapy in patients with stage IV NSCLC: Final analysis of phase II, open label, randomized, multicenter trial. J Clin Oncol (2019) 37:9039. 10.1200/JCO.2019.37.15_suppl.9039

[110] Demaria S Formenti SC. The abscopal effect 67 years later: from a side story to center stage. Br J Radiol (2020) 93:20200042. 10.1259/bjr.20200042 32101479 PMC7217574

[111] Demaria S Ng B Devitt ML Babb JS Kawashima N Liebes L. Ionizing radiation inhibition of distant untreated tumors (abscopal effect) is immune mediated. Int J Radiat Oncol Biol Phys (2004) 58:862–70. 10.1016/j.ijrobp.2003.09.012 14967443

[112] Ferrara TA Hodge JW Gulley JL. Combining radiation and immunotherapy for synergistic antitumor therapy. Curr Opin Mol Ther (2009) 11:37–42. 19169958 PMC3474202

[113] Akutsu Y Matsubara H Urashima T Komatsu A Sakata H Nishimori T. Combination of direct intratumoral administration of dendritic cells and irradiation induces strong systemic antitumor effect mediated by GRP94/gp96 against squamous cell carcinoma in mice. Int J Oncol (2007) 31:509–15. 17671676

[114] Teitz-Tennenbaum S Li Q Rynkiewicz S Ito F Davis MA McGinn CJ. Radiotherapy potentiates the therapeutic efficacy of intratumoral dendritic cell administration. Cancer Res (2003) 63:8466–75. 14679011

[115] Chi KH Liu SJ Li CP Kuo HP Wang YS Chao Y. Combination of conformal radiotherapy and intratumoral injection of adoptive dendritic cell immunotherapy in refractory hepatoma. J Immunother (2005) 28:129–35. 10.1097/01.cji.0000154248.74383.5e 15725956

[116] Finkelstein SE Iclozan C Bui MM Cotter MJ Ramakrishnan R Ahmed J. Combination of External Beam Radiotherapy (EBRT) With Intratumoral Injection of Dendritic Cells as Neo-Adjuvant Treatment of High-Risk Soft Tissue Sarcoma Patients. Int J Radiat OncologyBiologyPhysics (2012) 82:924–32. 10.1016/j.ijrobp.2010.12.068 PMC4241354 21398051

[117] Wang C Pu J Yu H Liu Y Yan H He Z. A Dendritic Cell Vaccine Combined With Radiotherapy Activates the Specific Immune Response in Patients With Esophageal Cancer. J Immunother (2017) 40:71–6. 10.1097/CJI.0000000000000155 28125513

[118] Versteven M Van den Bergh JMJ Marcq E Smits ELJ Van Tendeloo VFI Hobo W. Dendritic Cells and Programmed Death-1 Blockade: A Joint Venture to Combat Cancer. Front Immunol (2018) 9:394. 10.3389/fimmu.2018.00394 29599770 PMC5863527

[119] Beltra JC Manne S Abdel-Hakeem MS Kurachi M Giles JR Chen Z. Developmental Relationships of Four Exhausted CD8(+) T Cell Subsets Reveals Underlying Transcriptional and Epigenetic Landscape Control Mechanisms. Immunity (2020) 52:825–41.e8. 10.1016/j.immuni.2020.04.014 32396847 PMC8360766

[120] Dammeijer F van Gulijk M Mulder EE Lukkes M Klaase L van den Bosch T. The PD-1/PD-L1-Checkpoint Restrains T cell Immunity in Tumor-Draining Lymph Nodes. Cancer Cell (2020) 38:685–700.e8. 10.1016/j.ccell.2020.09.001 33007259

[121] Mohsenzadegan M Peng RW Roudi R. Dendritic cell/cytokine-induced killer cell-based immunotherapy in lung cancer: What we know and future landscape. J Cell Physiol (2020) 235:74–86. 10.1002/jcp.28977 31222740

[122] Wilgenhof S Corthals J Heirman C van Baren N Lucas S Kvistborg P. Phase II Study of Autologous Monocyte-Derived mRNA Electroporated Dendritic Cells (TriMixDC-MEL) Plus Ipilimumab in Patients With Pretreated Advanced Melanoma. J Clin Oncol (2016) 34:1330–8. 10.1200/JCO.2015.63.4121 26926680

[123] Verma V Shrimali RK Ahmad S Dai W Wang H Lu S. PD-1 blockade in subprimed CD8 cells induces dysfunctional PD-1(+)CD38(hi) cells and anti-PD-1 resistance. Nat Immunol (2019) 20:1231–43. 10.1038/s41590-019-0441-y PMC7472661 31358999

[124] Routy B Le Chatelier E Derosa L Duong CPM Alou MT Daillère R. Gut microbiome influences efficacy of PD-1–based immunotherapy against epithelial tumors. Science (2018) 359:91–7. 10.1126/science.aan3706 29097494

